# Supplementary material for: Hydrodynamic regimes modulate nitrogen fixation and the mode of diazotrophy in Lake Tanganyika
Source: Nat Commun. 2023 Oct 18;14:6591. doi: 10.1038/s41467-023-42391-3 (PMC10584864; doi:10.1038/s41467-023-42391-3)
Supplement: Supplementary file 1 — Supplementary Information [file 41467_2023_42391_MOESM1_ESM.pdf]

Supplementary Information for

Hydrodynamic regimes modulate nitrogen fixation and the mode of diazotrophy in Lake Tanganyika

Benedikt Ehrenfels, Kathrin B.L. Baumann, Robert Niederdorfer, Athanasio S. Mbonde, Ismael A. Kimirei, Thomas Kuhn, Paul M. Magyar, Daniel Odermatt, Carsten J. Schubert, Helmut Bürgmann, Moritz F. Lehmann, Bernhard Wehrli, Cameron M. Callbeck*

*Corresponding author. Email: cameron.callbeck@unibas.ch

This PDF file includes:

Supplementary Discussion
Supplementary Methods
Supplementary Figs. 1-9
Supplementary Tables 1-8

Supplementary Discussion

Rates of nitrogen removal in Lake Tanganyika

In line with previous nitrogen loss surveys in Lake Tanganyika[1,2], we measured anammox and denitrification rates from ^{15}N addition experiments (^{15}N-NO_3^-, ^{15}N-NO_2^-, and ^{15}N-NH_4^+) in our 2018 north-south transect (Supplementary Fig. 7). Anammox activity, which is indicated by the production of $^{29}N_2$ gas from ^{15}N-NH_4^+ + ^{14}N-NO_2^- and ^{15}N-NO_2^- + ^{14}N-NH_4^+ additions, ranged from below the limit of detection to 147 nM N d^{-1} (Fig. S7b). These values are within the same range as previously quantified by Schubert et al.[1], but three times higher than the maximum anammox rates reported by Callbeck et al.[2]. Rates of denitrification, which were identified based on the generation of $^{30}N_2$ gas from ^{15}N-NO_3^-, attained up to 102 nM N d^{-1} but were found only at Station 4 (120 m depth; Supplementary Fig. 7a). Latitudinally, our volumetric rates of N removal via anammox and denitrification were greatest in the northern and central basins of Lake Tanganyika (max. 147 nM N d^{-1}), while the lowest rates were measured in the southern basin (max. 32.2 nM N d^{-1}; Supplementary Fig. 7c), consistent with our previous study[2]. Congruently, the depth-integrated N removal rates (0-175 m) were significantly higher at stations 2-6 in the north/center (1.0-4.6 mmol N m^{-2} d^{-1}) compared to stations 7-9 in the south basin (0.3-1.3 N m^{-2} d^{-1}; Supplementary Fig. 7f).

N_2 fixation and nitrogen loss

The input of fixed N, via N fixation near the surface and within the anoxic zone in Lake Tanganyika, potentially places important limits on nitrogen loss via anammox and denitrification in anoxic waters. In support of this argument, we find a striking correlation between the depth-integrated rates of N removal and N fixation (Spearman, one-sided, R^2 = 0.7, $p < 0.05$; Supplementary Fig. 8). Moreover, anammox rates were three times higher in

2018 compared to the same season in 2019[2], when filamentous cyanobacteria were absent and surface N fixation was <1 nM N d^{-1} Supplementary Fig. 6). The amount of exported organic N arriving in anoxic depths cannot be inferred from our data, due to organic matter degradation and subsequent nitrification of sinking surface particles in the oxic waters in between[2] (as indicated by the NO_3^- concentration maximum and $\delta^{15}N$-NO_3^- minimum at ~100 m; Supplementary Fig. 4). However, elevated turbidity and high abundances of *Dolichospermum* observed in waters below the surface bloom indicate that a significant fraction of the fixed N from cyanobacteria was exported to anoxic waters (Fig. 3f and Supplementary Fig. 1d). Enhanced organic N export linked to blooms of diazotrophic cyanobacteria was previously observed in the oligotrophic open ocean[3–5]. Such pulses of organic matter were identified to enhance anammox rates[6] and act as a driver behind the patchy occurrence of denitrification in marine environments[7,8]. In line with these observations, the elevated rates of anammox and denitrification compared to 2019 may be linked to the bloom of filamentous cyanobacteria in the euphotic zone above.

We suggest that the export of freshly-fixed N from surface waters, coupled with enhanced organic matter remineralization of sinking organic matter in the anoxic chlorophyll maximum (ACM)[2], contributed to the enhanced rates of N loss in the northern basin.

Dolichospermum and *Chlorobium*

Overall, *Dolichospermum* and *Chlorobium* share in common a general autotrophic based physiology, a reliance on light, as well as a capacity for vertical migration (via buoyancy) enabling some degree of navigation in the stratified water column. These general features distinguish these members from the heterotrophic diazotrophs identified at Station 7 (discussed below). How *Dolichospermum* and *Chlorobium* differ can be ascribed to the energy demands needed to support growth in the vertical water column (i.e., primary

chlorophyll maximum (PCM) vs ACM niches). For example, the *Chlorobium* metagenome is more specialized towards maximizing energy efficiency compared to *Dolichospermum*, which likely reflects the extreme energetic limitations encountered in the ACM versus the PCM. Some of these key ATP-saving measures include the use of the reverse tricarboxylic acid cycle (rTCA) over the Calvin-Benson-Bassham cycle (CBB) carbon fixation pathways (Supplementary Tables 3 and 4). The rTCA cycle encoded in *Chlorobium* (Fig. 3d), is commonly identified in anoxic microbes due to its higher energy efficiency (i.e., molecules of CO_2 fixed per mole of ATP utilized[9]). In addition, Lake Tanganyika *Chlorobium* had a streamlined genome (half the size of other diazotrophs), a reduced metabolic plasticity, and a reduced capacity for amino acid catabolism (20-86% reduction compared to other diazotrophs) (Fig. 3b). The employment of such ATP-saving measures could afford *Chlorobium* a higher degree of ATP allocation towards N_2 fixation – another ATP-demanding process. Apart from N_2 fixation, both *Dolichospermum* and *Chlorobium* are also likely contributors to the measured rates of carbon fixation in the PCM and ACM measured previously[2].

Aquabacterium and *Pseudomonas*

Aquabacterium and *Pseudomonas* indicated a strong dependence on organic carbon for growth. Encoded in their genomes were genes associated with the uptake (e.g., ATP binding cassette; ABC), sensing (e.g., methyl accepting chemotaxis proteins) and degradation (e.g., aminopeptidases) of amino acid/polypeptides (Supplementary Tables 5 and 6). Specifically, both members had ABC transport systems for branched-chain amino acids (isoleucine, leucine and valine) and oligopeptides, including downstream degradation pathways. Similarly, marine studies have found that *Pseudomonas* species generally dominate amino acid degradation[10]. *Pseudomonas*, additionally contained pathways for the uptake and

utilization of monosaccharides (e.g., maltose and maltodextrin), as well as methyl accepting chemotaxis proteins used for detecting sugars (ribose and galactose) in the environment.

Other salient features of both *Aquabacterium* and *Pseudomonas* include several genes encoding for TonB-dependent receptors; they comprise 13 and 9 total annotated receptors (Fig. 3b and Supplementary Tables 5 and 6), respectively. TonB-dependent receptors facilitate the uptake of high molecular weight compounds (>600 Da), such as dissolved organic matter and aromatic substrates[11]. The putative uptake of DOM by *Aquabacterium* and *Pseudomonas* was also accompanied by pathways and enzymes (e.g., mono-/di-oxygenases) that specialize in the decomposition of high molecular weight compounds, such as benzoate, among other compounds. *Aquabacterium* contained a pathway for benzoate degradation. Benzoate degradation was also detected in *Pseudomonas*, alongside an even larger array of mono-/di-oxygenases involved in biphenyl, n-phenylalkanoic, chloroaromatic degradation, including downstream pathways involved in the processing of intermediate degradation products, such as catechol, salicylate, gentisate, protocatechuate, and homogentisate.

Supplementary Methods

Metagenomic pipeline analysis

The pipeline has been reported in Ehrenfels et al.[12]. Briefly, the quality of metagenomic reads was assessed using FastQC (version 0.12.1) (http://www.bioinformatics.babraham.ac.uk/projects/fastqc). Prinseq[13] was used to trim the metagenomics reads (minimum quality mean 20) if not meeting the quality requirements. The taxonomic assignment was performed with Kaiju (version 1.9.2)[14]. Each sample was assembled using Megahit (version 1.2.9) with the options meta-sensitive and –min_contig length 500[15] and reads were mapped back using BBmap[16] to assess the coverage of the raw reads on the contigs. The bam files were converted using sambamba (GPL v2+)[17] and

SAMtools (version 1.18)[18], and the reads were subsequently counted using the feature counts package from subread 1.6.4[19]. For functional annotation of contigs, we used prokka (version 1.13)[20]. Afterwards, we extracted the genes for N fixation (nifHDK) from the gtf file. The quantified genes were normalized for the gene lengths and sequencing depth. The sequencing depth, filtered reads, mean contig length, and mapped reads for each sample are summarized in Supplementary Table 1.

High-quality trimmed reads from all sampling depths (n = 15) were co-assembled into scaffolds using Megahit[15] with the options --min_contig 1000 and the kmer-length 21,37,47,71,87,95. The metaWRAP (version 1.3)[21] binning and refinement modules were applied to the co-assembly to recover high-quality metagenome assembled genomes (MAGs). Completeness and contamination rates of the final MAGs were assessed using CheckM (version 1.1.6)[22]. We only used MAGs that passed a threshold for completion of 50 %, and contamination rates less than 10 % following the established standards[23]. Taxonomical classification of high-quality MAGs was performed via the gtdbtk classify pipeline[24]. MAG abundances were assessed using coverM (version 0.2.0). Here, raw reads were mapped against the putative genomes, and abundance is expressed as the coverage of raw reads on the MAG. The total number of MAGs recovered from our samples, along with completeness, and contamination are summarized in Supplementary Table 2.

N_2 fixation genes from all MAGs were concatenated, and aligned and a maximum likelihood tree was constructed with MEGAX (version 11.0.13) using the standard parameters and 1000 times bootstrapping[25].

Stable isotope analysis of nitrate and ammonium

Samples for nitrate and ammonium isotope samples from 2018 were sterile-filtered (0.2 µm), stored at ~4 °C, and frozen upon return to land until analysis. Traces of nitrite, where present,

were removed with 1 mL sulfamic acid (1 %) per 50 mL sample[26]. The natural abundance N isotopic composition of nitrate was determined with the denitrifier method[27,28]. Briefly, 10-30 nanomoles of sample nitrate were transformed into nitrous oxide by a cultured strain of denitrifying bacteria (*Pseudomonas chlororaphis* ssp. *aureofaciens*, ATCC 13985). The $\delta^{15}N$ of the resulting nitrous oxide was measured on an isotope ratio mass spectrometer (DELTA V Plus, Thermo Fisher Scientific, Bremen, Germany; software ISODAT3.0 version 3.0.94.12) coupled to a customized purge-and-trap system modified after McIlvin and Casciotti[29]. Sample $\delta^{15}N$ was determined by calibration with internal and international KNO_3 reference materials with $\delta^{15}N$ values of 14.15 ‰ (internal UBN-1), 4.7 ‰ (IAEA-NO-3), and 1.8 ‰ (USGS 34), respectively. Replicate reproducibility was generally better than 0.3‰.

The natural abundance N isotopic composition of ammonium was quantified by a combination of methods[30,31]. Briefly, >100 nanomoles of sample ammonium were transformed to gaseous ammonia by adding MgO. The ammonia was trapped as ammonium on pre-combusted and acidified glass fiber discs (Whatman #1823010), sealed between two Teflon membranes (Millipore LCWP 0130 0). The trapped ammonium was then chemically oxidized to nitrate by the addition of ultrapure sodium persulfate solution. The pH was adjusted to 4 and the N isotopic composition of the resulting nitrate was determined with the denitrifier method as described above. Sample isotope ratios were quantified using a two-step calibration. UBN-1, IAEA-NO-3, and USGS34 were used as standards for the denitrifier method, whereas the international standards USGS-25, IAEA-N-1, and IAEA-N-2 with known $\delta^{15}N$ values of -30.4 ‰, 0.4 ‰, and 20.3 ‰, respectively, were used for the correction of processing/blank effects of the persulfate steps. Replicate reproducibility was typically ±1.0 ‰.

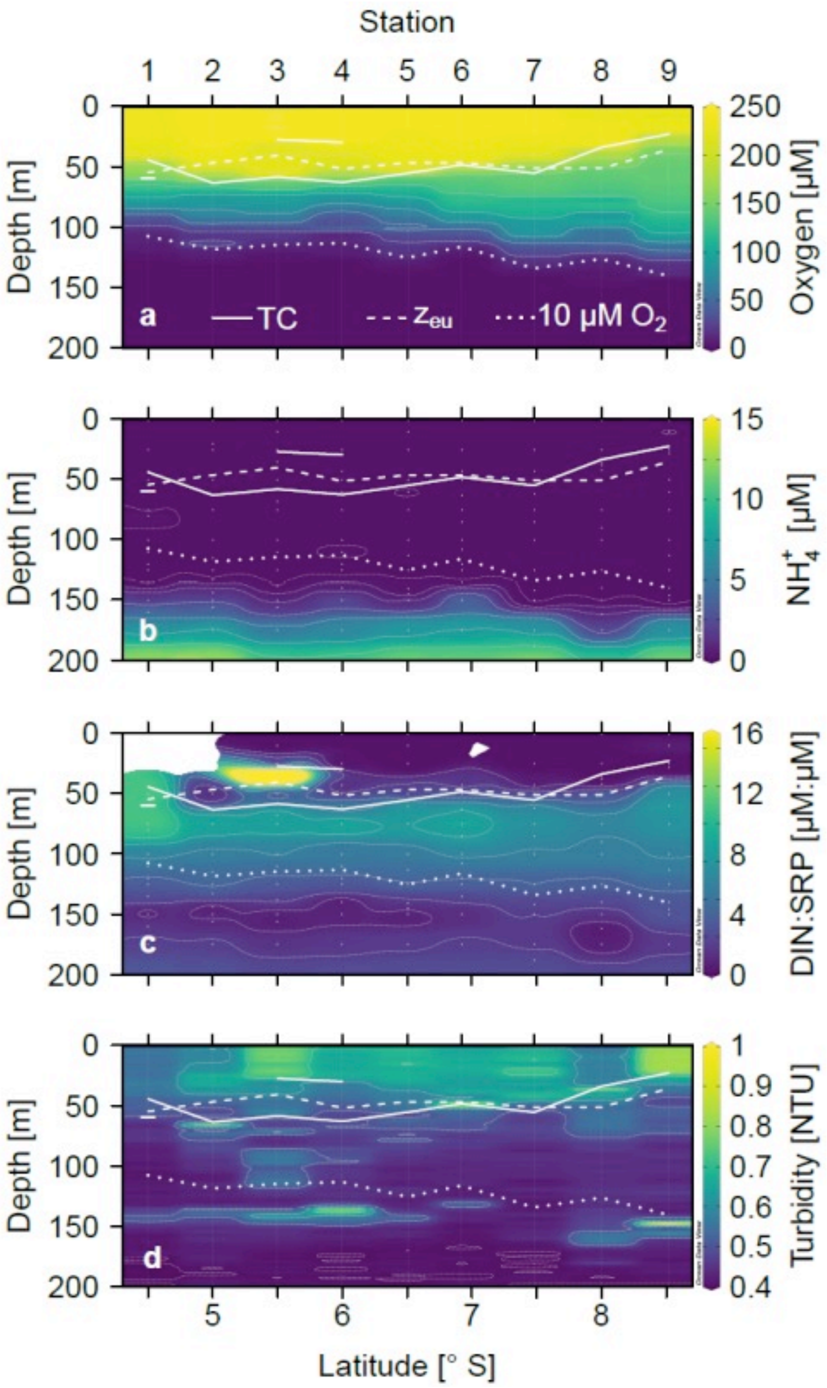

Supplementary Fig. 1. Distribution of (a) oxygen, (b) ammonium (NH_4^+), (c) the molar ratio of dissolved inorganic nitrogen (DIN) to soluble reactive phosphate (SRP), and (d) turbidity in Lake Tanganyika in April/May 2018. The solid white line depicts the position of the thermocline (TC), the dashed white line represents the euphotic depth (z_{eu}), and the dotted white line indicates the 10 µM oxygen (O_2) boundary. Dots (discrete samples) or vertical lines (CTD profiles) mark the sampling depths.

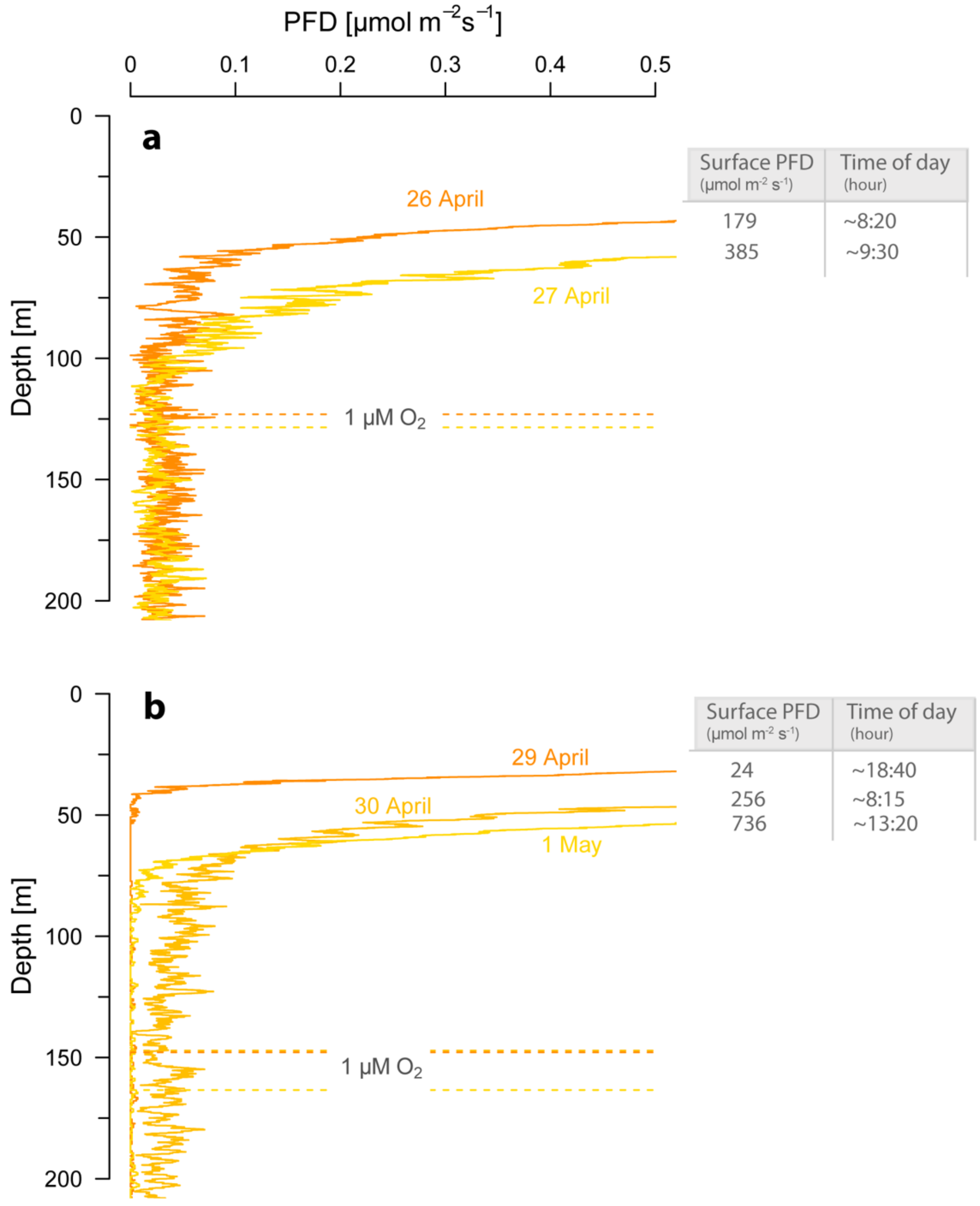

Supplementary Fig. 2. Vertical light irradiance profiles for Lake Tanganyika. The light irradiance values are expressed as the photon flux density (PDF) for Stations 2 and 7, which are indicated in panels (a) and (b), respectively. The corresponding surface light irradiance values, and the time of day when the measurements were performed are indicated in the adjacent table. Data are from the April-May 2019 campaign and were previously shown in Callbeck et al.[2] (licensed under a Creative Commons Attribution 4.0: https://creativecommons.org/licenses/by/4.0/).

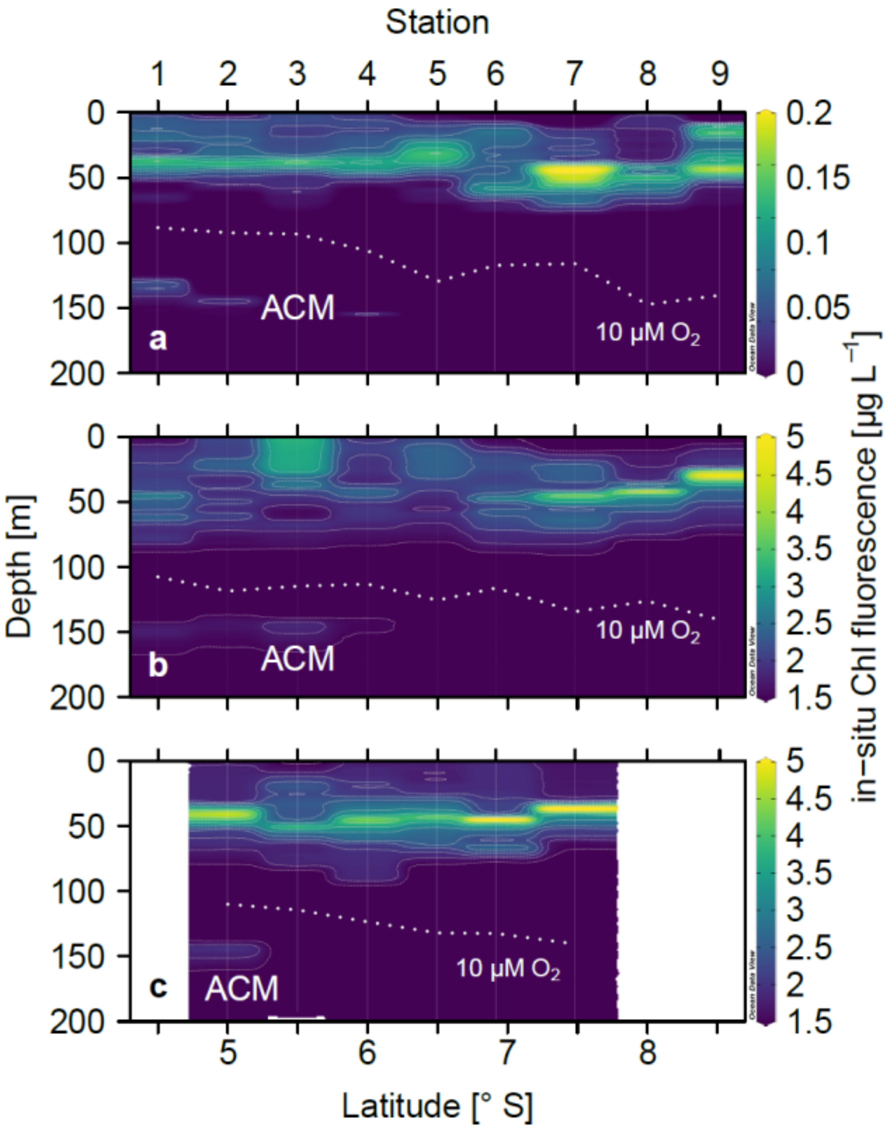

Supplementary Fig. 3. In-situ chlorophyll fluorescence distribution showing, besides the omnipresent primary chlorophyll maximum in surface waters (PCM), the presence of an anoxic chlorophyll maximum (ACM) at ~150 m in the northern and central basins during all three sampling campaigns: (a) September/October 2017, (b) April/May 2018, (c) April/May 2019. White vertical lines indicate sampling stations, and the dotted line depicts the 10 µM oxygen boundary. Note that the uncalibrated in-situ chlorophyll fluorescence values were used.

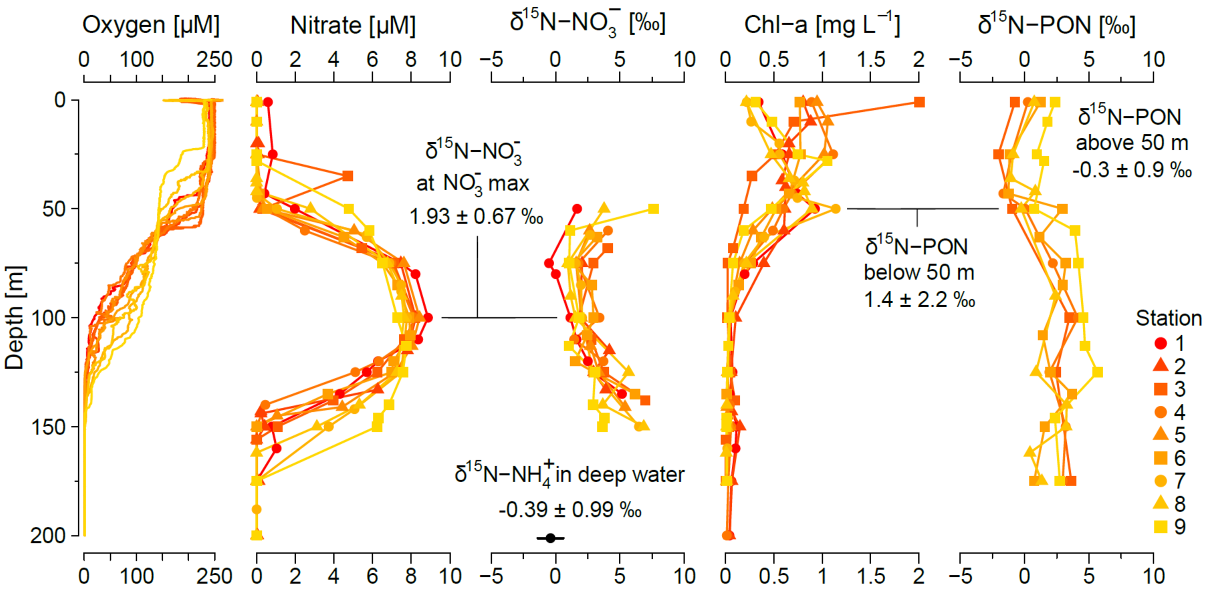

Supplementary Fig. 4. Vertical profiles of dissolved oxygen, nitrate (NO_3^-), $\delta^{15}N$-NO_3^-, chlorophyll-a, and the $\delta^{15}N$ of particulate organic nitrogen (PON) in Apr/May 2018. Hypolimnetic $\delta^{15}N$-NH_4^+ data are plotted in the $\delta^{15}N$-NO_3^- panel as mean value ± standard deviation.

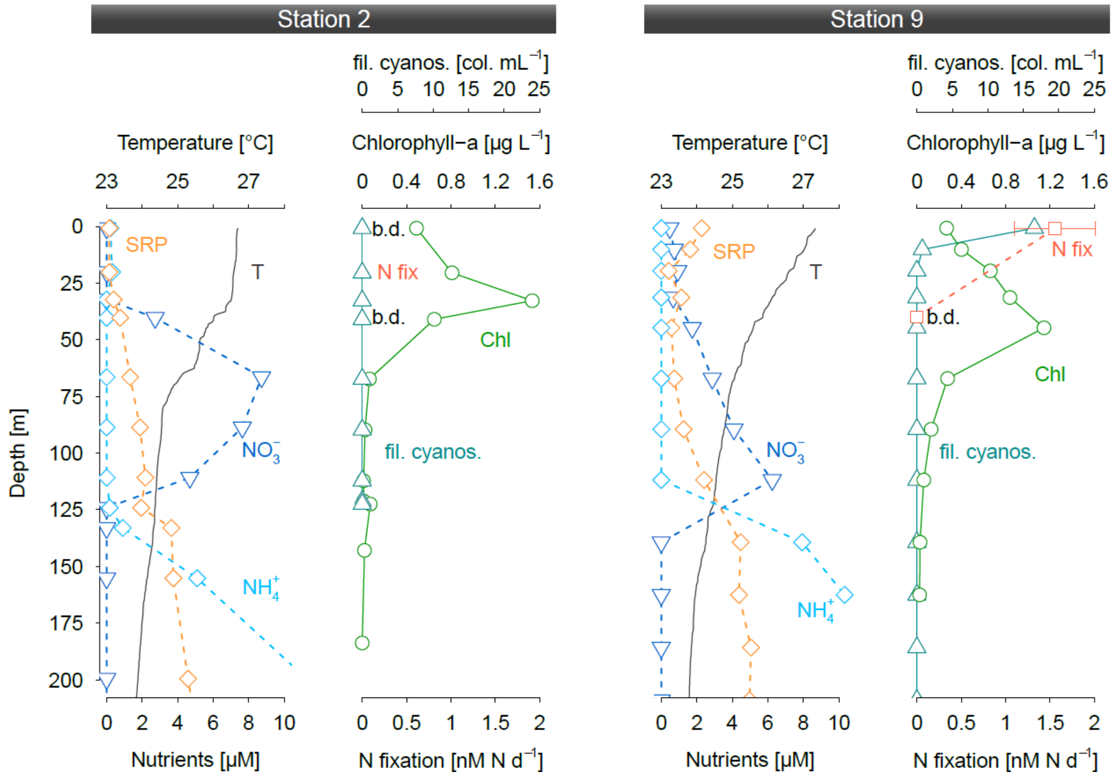

Supplementary Fig. 5. Vertical profiles of temperature, nutrient concentrations, chlorophyll-*a*, abundance of filamentous cyanobacteria, and nitrogen fixation rates at Stations 2 (north) and 9 (south) in September/October 2017. N_2 fixation rates are presented as mean values of duplicate samples, with error bars representing the standard deviation. b.d. = below detection limit.

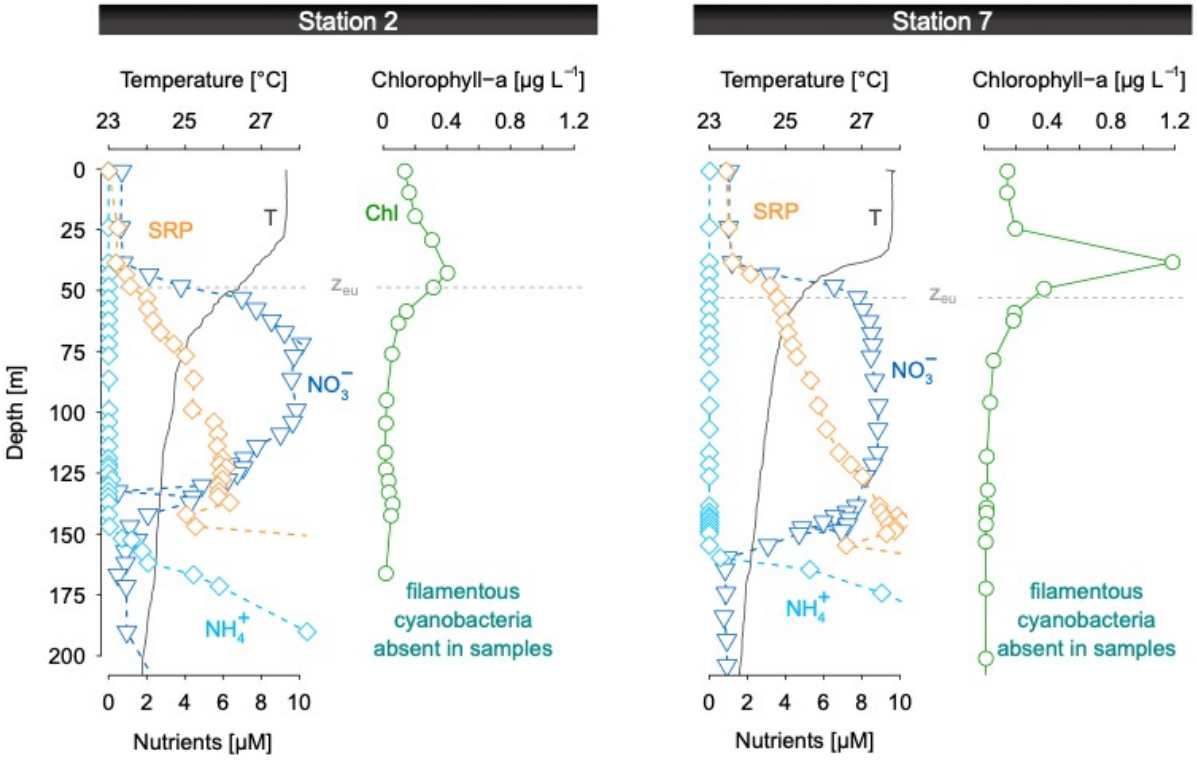

Supplementary Fig. 6. High-resolution profiles of temperature, nutrient concentrations, and chlorophyll-a, at the main sampling stations 2 (north) and 7 (south) in Apr/May 2019. z_{eu} marks the lower boundary of the euphotic zone (i.e., 1 % of the surface irradiance). At both stations, filamentous, nitrogen-fixing cyanobacteria were absent.

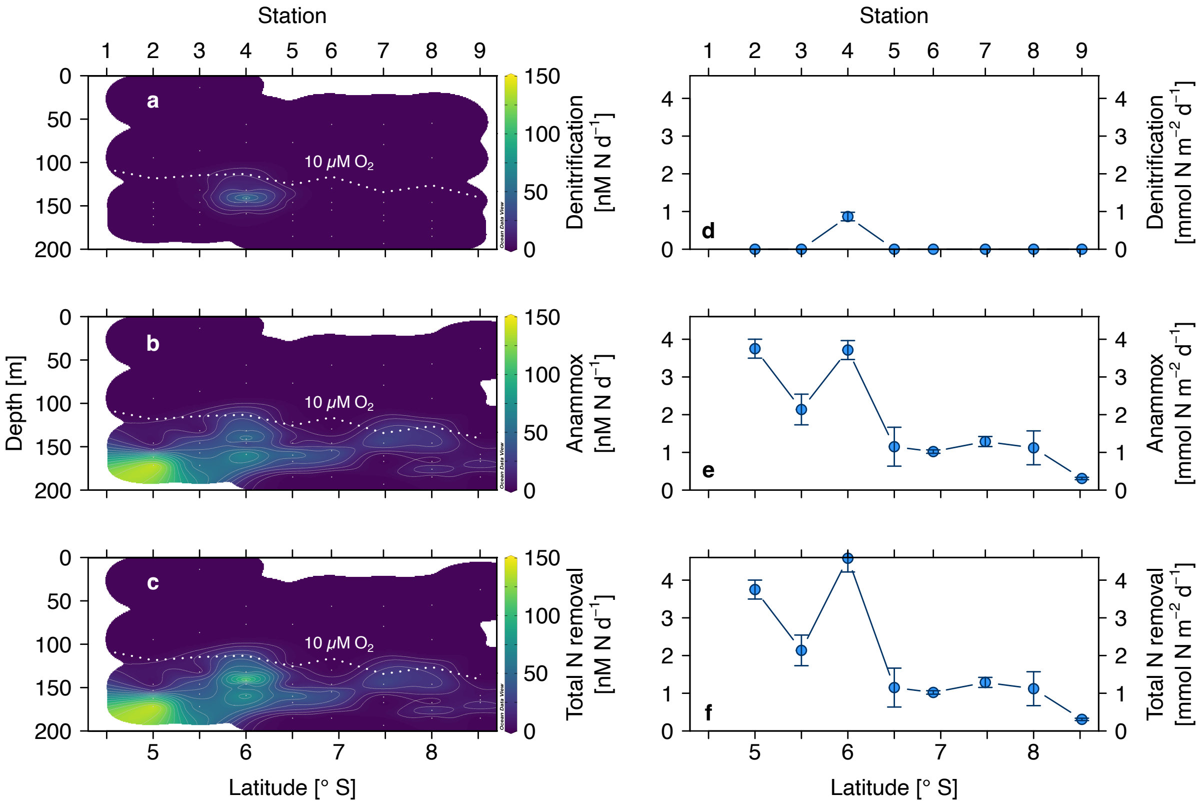

Supplementary Fig. 7. Distribution of nitrogen removal rates in Lake Tanganyika in April/May 2018. (a) distribution and (d) depth-integrated values of denitrification (0-175 m); (b) distribution and (e) depth-integrated values of anammox (0-175 m); (c) distribution and (f) depth-integrated values of total N removal (0-175 m). Dots (discrete samples) or vertical lines (CTD profiles) mark the sampling depths. The slope of the linear regression was used to calculate the rates of N_2 production as a function of time (in panels d-f), and the standard error was derived from the deviation in the linear slope over the five time points.

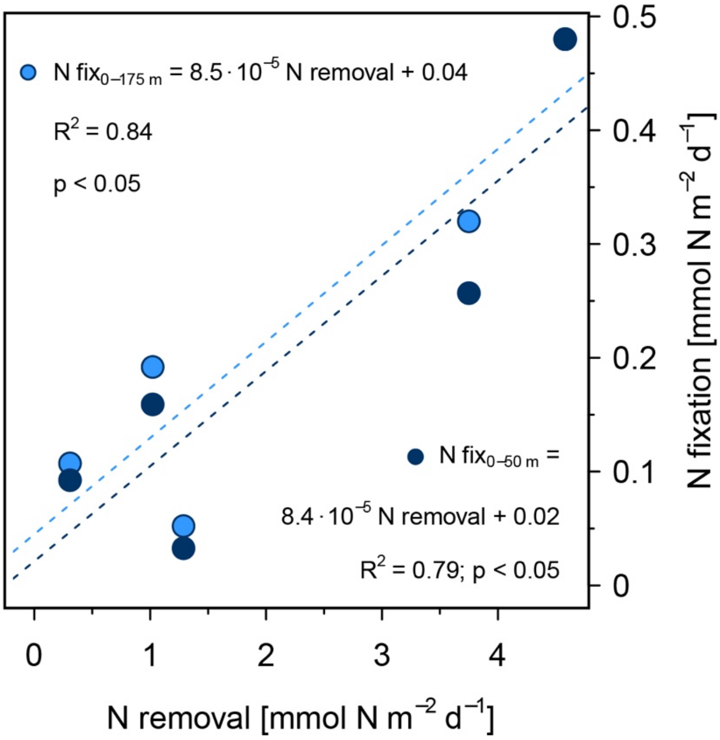

Supplementary Fig. 8. Depth-integrated nitrogen fixation rates versus nitrogen removal rates (denitrification plus anammox) in April/May 2018. A linear regression line is shown with R^2 and p-values (Spearman correlation test, one-sided, and a 95% confidence interval).

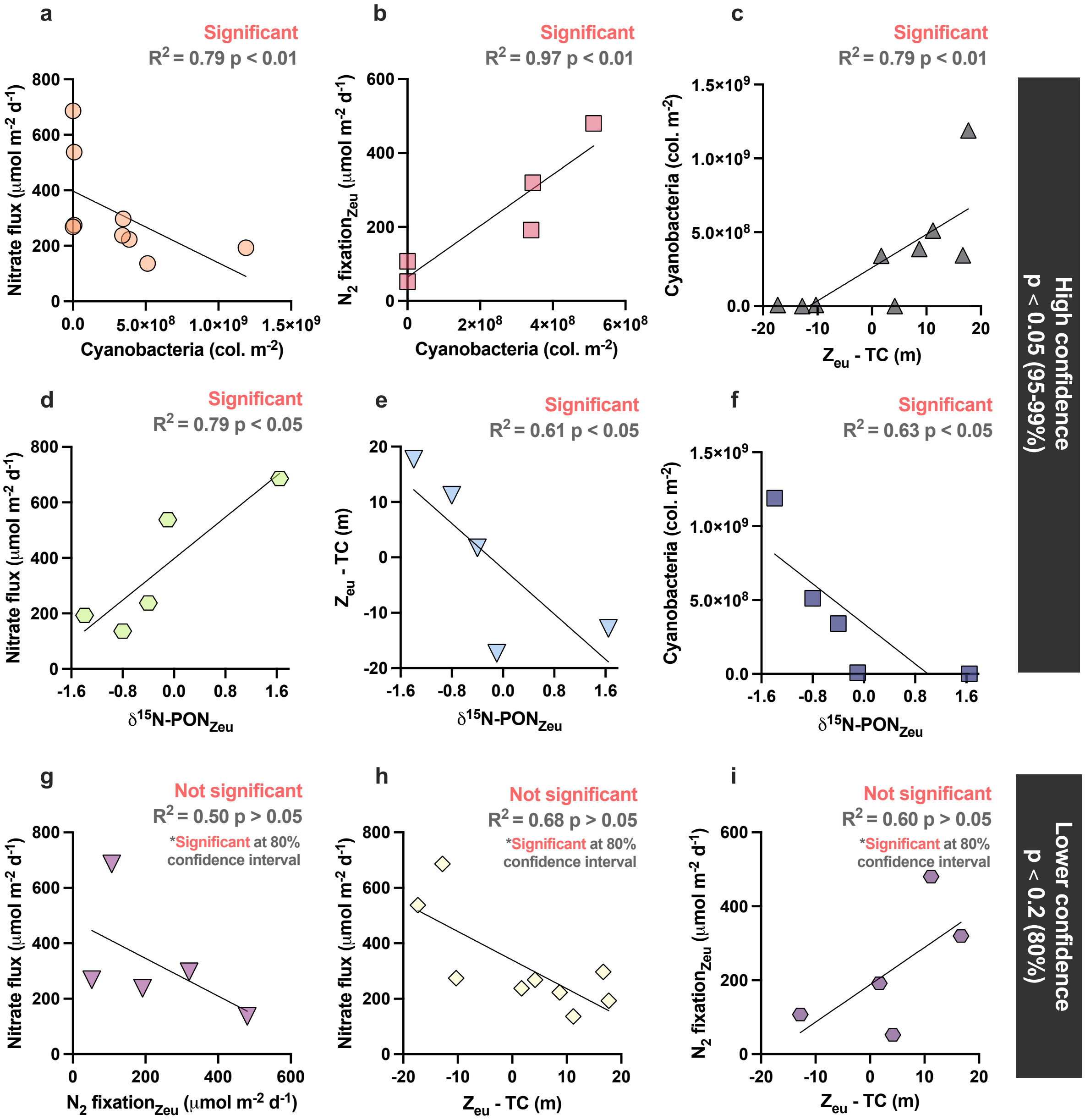

Supplementary Fig. 9. Correlation matrix with the integrated rates of N_2 fixation (panels b, g, i; 0-43 m; N_2 fixation$_{zeu}$), the δ^{15}N of particulate organic nitrogen in the euphotic zone (panels d, e, f; 0-43 m; δ^{15}N-PON$_{zeu}$), the abundance of filamentous, diazotrophic cyanobacteria (panels a, c, f), the distance between the euphotic depth (Z_{eu}) and the thermocline (Z_{eu} - TC) as well as the estimated nitrate flux (panels a, d, h; NO_3^- flux). A linear regression line is shown with R^2 and p-values (Spearman correlation test, one-sided) indicated above each panel. Note, that performing a correlation analysis between δ^{15}N-PON$_{zeu}$ versus volumetric N_2 fixation rates is not possible because these measurements were not always sampled at the same station (only three stations overlap). Correlative analyses that are statistically significant at a 95-99% ($p < 0.05$), and 80% confidence interval ($p \leq 0.2$) are indicated.

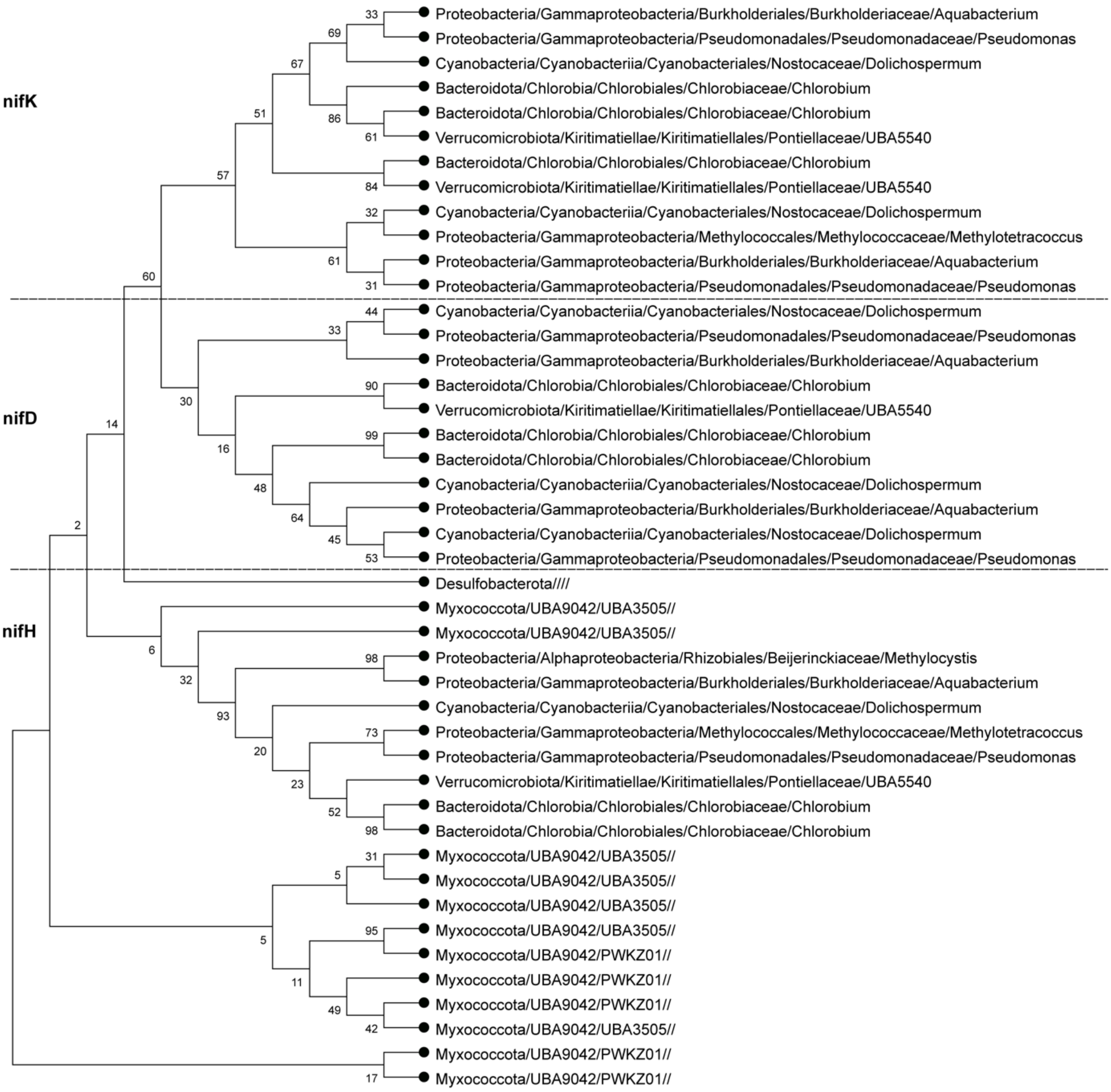

Supplementary Fig. 10. Phylogenetic diversity of recovered *nifHDK* gene sequences. The unrooted tree was calculated using the maximum likelihood method (bootstraps = 1000). The phylogenetic *nifH* tree is shown in Fig. 3a.

Supplementary Table 1. Sequencing depth, filtered reads, mean contig length, mapped reads, and nitrogenase gene abundance (*nifHDK*) for each metagenome sample.

Sample name	Station	Depth (m)	Sequcences No	Filtered sequences No	Contig No	N50	Mapping rate (%)	*nif* genes (gpm)
TM1	2	1	45'945'846	45'850'634	349'736	1'897	81	76.4
TM2	2	20	32'691'725	32'612'128	282'763	2'045	77	71.5
TM3	2	75	35'717'635	35'654'433	370'836	1'855	77	12.0
TM4	2	125	54'104'248	53'894'203	424'938	2'000	80	21.4
TM5	2	133	38'831'136	38'773'808	363'699	2'167	81	16.6
TM6	2	144	44'452'814	44'417'375	421'008	2'452	79	248.3
TM7	2	156	35'749'570	49'043'134	484'366	1'691	72	247.9
TM8	7	1	49'367'533	49'302'851	301'447	2'244	80	80.8
TM9	7	43	39'774'511	39'701'865	341'047	1'779	76	75.4
TM10	7	62	42'017'178	41'940'222	432'646	1'941	80	26.7
TM11	7	85	49'043'134	48'934'588	464'353	2'483	80	13.1
TM12	7	125	39'440'673	39'261'389	368'429	1'939	77	14.5
TM13	7	142	41'273'865	41'196'218	367'824	2'334	81	19.0
TM14	7	150	38'741'005	38'659'973	407'200	2'140	78	42.9
TM15	7	188	55'904'770	55'786'538	268'774	1'172	57	62.9

Supplementary Table 2. Metagenome assembled genomes (MAGs) of potential diazotrophs recovered from our samples, including bin completeness, contamination, and genome size. MAGs shown in bold represent key marker species discussed in-text and in Fig. 3.

MAG_ID	Phylum	Class	Order	Family	Genus	Completeness [%]	Contamination	GC [%]	N50	Genome size [kbp]
TAN_361	Actinobacteriota	Acidimicrobiia	IMCC26256	IMCC26256	NA	98.29	1.377	71	55957	2961.4
TAN_385	Actinobacteriota	Acidimicrobiia	IMCC26256	PALSA-555	NA	98.29	1.377	68	116468	2744.2
TAN_86	Actinobacteriota	Thermoleophilia	UBA2241	UBA2241	UBA6103	94.82	1.58	70	10610	3355.7
**TAN_426**	**Bacteroidota**	**Chlorobia**	**Chlorobiales**	**Chlorobiaceae**	**Chlorobium**	**99.44**	**0.276**	**55**	**61882**	**2165.3**
TAN_465	Bacteroidota	Chlorobia	Chlorobiales	Chlorobiaceae	Chlorobium	95.02	2.651	53	38304	2186.0
TAN_342	Chloroflexota	Dehalococcoidia	UBA2979	UBA2979	NA	58.39	1.98	70	9293	1275.7
TAN_360	Chloroflexota	Chloroflexia	Chloroflexales	Roseiflexaceae	UBA965	83.64	0.943	54	40551	2517.3
TAN_471	Chloroflexota	Dehalococcoidia	UBA2979	UBA2979	NA	58.99	3.245	70	3007	1458.8
TAN_77	Chloroflexota	Dehalococcoidia	UBA2979	UBA2979	NA	92.24	0.11	72	30246	1918.5
TAN_81	Chloroflexota	Dehalococcoidia	UBA2979	UBA2979	NA	95.04	5.06	73	54921	2978.5
**TAN_219**	**Cyanobacteria**	**Cyanobacteriia**	**Cyanobacteriales**	**Nostocaceae**	**Dolichospermum**	**94.11**	**0.851**	**37**	**8463**	**3866.0**
TAN_118	Desulfobacterota	Binatia	UBA9968	UBA9968	NA	98.68	2.903	58	90406	5532.9
TAN_143	Desulfobacterota	Syntrophia	Syntrophales	UBA5619	UBA5619	65.51	1.724	58	43496	3216.4
TAN_182	Desulfobacterota	Binatia	UBA12015	NA	NA	96.64	4.569	71	57992	6242.4
TAN_251	Desulfobacterota	Binatia	Bin18	Bin18	NA	92.43	3.361	53	24727	6119.9
TAN_254	Desulfobacterota	Binatia	UBA12015	NA	NA	96.64	3.87	69	33986	6213.7
TAN_257	Desulfobacterota	Binatia	UBA9968	UBA9968	DP-20	85.62	3.248	59	5263	5013.7
TAN_292	Desulfobacterota	Desulfobaccia	Desulfobaccales	0-14-0-80-60-11	0-14-0-80-60-11	96.45	0.806	59	20357	2986.9
TAN_309	Desulfobacterota	BSN033	BSN033	UBA1163	RBG-16-49-23	70.55	7.652	47	3733	3378.4
TAN_35	Desulfobacterota	BSN033	SM23-61	SM23-61	NA	95.48	4.516	51	13343	4951.3
TAN_351	Desulfobacterota	Syntrophia	Syntrophales	UBA5619	UBA5619	70.68	0	62	29332	3259.8
TAN_373	Desulfobacterota	Binatia	HRBIN30	NA	NA	80.71	8.168	71	4809	4988.2

Supplementary Table 2 (continued).

MAG_ID	Phylum	Class	Order	Family	Genus	Completeness [%]	Contamination	GC [%]	N50	Genome size [kbp]
TAN_424	Desulfobacterota	Desulfomonilia	Desulfomonilales	Desulfomonilaceae	NA	94.51	0.645	57	24727	4575.5
TAN_54	Desulfobacterota	BSN033	BSN033	UBA1163	RBG-16-49-23	81.22	1.935	47	10554	4025.8
TAN_63	Desulfobacterota	NA	NA	NA	NA	87.3	2.734	56	8974	2756.5
TAN_207	Myxococcota	Polyangia	Polyangiales	NA	NA	73.26	1.451	66	4484	3711.9
TAN_263	Myxococcota	Bradimonadia	NA	NA	NA	97.2	1.635	75	82918	5078.4
TAN_286	Myxococcota	UBA9042	UBA3505	NA	NA	88.56	1.505	73	21343	5744.7
TAN_305	Myxococcota	Polyangia	Polyangiales	Polyangiaceae	NA	54.22	3.817	69	3261	2485.3
TAN_337	Myxococcota	Polyangia	Polyangiales	Polyangiaceae	NA	86.55	3.387	69	20759	5877.3
TAN_422	Myxococcota	UBA9160	UBA9160	UBA6930	NA	89.13	2.58	70	97729	4444.3
TAN_457	Myxococcota	Polyangia	Polyangiales	Polyangiaceae	SYFR01	78.8	2.688	69	5226	3778.9
TAN_459	Myxococcota	UBA9042	PWKZ01	NA	NA	84.3	2.339	66	13934	6144.5
TAN_481	Myxococcota	Bradimonadia	UBA7976	UBA1532	NA	50.17	1.73	73	2321	2909.2
TAN_73	Myxococcota	Bradimonadia	UBA7976	UBA1532	NA	94.83	4.354	70	22155	5917.6
TAN_196	Omnitrophota	Koll11	GIF10	UBA6249	GCA-002774445	89.06	1.075	41	15985	1174.5
TAN_12	Planctomycetota	Phycisphaerae	Sedimentisphaerales	Anaerohalophaeraceae	PLanc-01	64.17	9.814	64	3358	6924.7
TAN_148	Planctomycetota	Planctomycetes	Pirellulales	UBA1268	F1-20-MAGs016	94.25	1.149	71	57588	4182.4
TAN_158	Planctomycetota	Brocadiae	Brocadiales	Brocadiaceae	Kuenenia	97.8	1.648	40	27426	3626.6
TAN_186	Planctomycetota	UBA1135	UBA1135	GCA-002686595	F1-120-MAGs118	54.38	0	70	4389	3705.6
TAN_386	Planctomycetota	UBA1135	UBA1135	GCA-002686595	GW928-bin9	83.28	2.15	70	16029	3579.5

Supplementary Table 2 (continued).

MAG_ID	Phylum	Class	Order	Family	Genus	Completeness [%]	Contamination	GC [%]	N50	Genome size [kbp]
TAN_316	Proteobacteria	Alphaproteobacteria	Rhizobiales	Beijerinckiaceae	Methylocystis	94.59	0.316	47	27303	2679.2
TAN_321	Proteobacteria	Alphaproteobacteria	Rhizobiales	Beijerinckiaceae	Methylocystis	94.31	3.375	64	16697	3192.6
TAN_323	Proteobacteria	Gammaproteobacteria	Burkholderiales	Burkholderiaceae	Aquabacterium	87.48	3.091	65	8733	2958.3
TAN_336	Proteobacteria	Alphaproteobacteria	Rhizobiales	Beijerinckiaceae	Methylocystis	94.53	2.584	62	16353	3373.5
TAN_350	Proteobacteria	Gammaproteobacteria	Methylococcales	Methylococcaceae	Methylotetracoccus	61.85	4.815	63	3104	2577.2
**TAN_398**	**Proteobacteria**	**Gammaproteobacteria**	**Burkholderiales**	**Burkholderiaceae**	**Aquabacterium**	**90.4**	**2.336**	**67**	**13698**	**4073.0**
TAN_410	Proteobacteria	Gammaproteobacteria	Methylococcales	Methylococcaceae	Methylotetracoccus	97.69	4.022	64	34269	4568.9
TAN_51	Proteobacteria	Gammaproteobacteria	Burkholderiales	Burkholderiaceae	Aquabacterium	70.41	2.383	68	4466	2907.1
TAN_70	Proteobacteria	Alphaproteobacteria	Rhizobiales	Beijerinckiaceae	Methylocystis	90.48	1.424	62	25399	2882.0
**TAN_78**	**Proteobacteria**	**Gammaproteobacteria**	**Pseudomonadales**	**Pseudomonadaceae**	**Pseudomonas_A**	**93.16**	**1.161**	**65**	**21835**	**4003.8**
TAN_449	Verrucomicrobiota	Kiritimatiellae	Kiritimatiellales	Pontiellaceae	UBA5540	63.57	1.576	51	3949	1635.9

Supplementary Table 3. Metagenome assembled genome (MAG) of *Dolichospermum* (TAN_219; Supplementary Table 2). Synthesis of the key genes and metabolic pathways presented in Fig. 3 and discussed in-text.

Gene	MAG contig	Start	Stop	Strand	Metabolic pathway
Nitrogenase FeMo-cofactor synthesis FeS core scaffold and assembly protein NifB	TAN_219_187	6072	7502	+	Nitrogen fixation
Cysteine desulfurase (EC 2.8.1.7), NifS subfamily	TAN_219_187	8035	9240	+	Nitrogen fixation
Iron-sulfur cluster assembly scaffold protein NifU	TAN_219_187	9627	10538	+	Nitrogen fixation
Nitrogenase (molybdenum-iron) reductase and maturation protein NifH	TAN_219_187	10813	11703	+	Nitrogen fixation
Nitrogenase (molybdenum-iron) alpha chain (EC 1.18.6.1)	TAN_219_187	11834	12760	+	Nitrogen fixation
Nitrogenase (molybdenum-iron) alpha chain (EC 1.18.6.1)	TAN_219_187	14640	15011	+	Nitrogen fixation
Nitrogenase (molybdenum-iron) beta chain (EC 1.18.6.1)	TAN_219_285	2444	909	-	Nitrogen fixation
Nitrogenase FeMo-cofactor scaffold and assembly protein NifE	TAN_219_301	52	873	+	Nitrogen fixation
Nitrogenase FeMo-cofactor scaffold and assembly protein NifN	TAN_219_301	1090	2418	+	Nitrogen fixation
hypothetical protein	TAN_219_301	2677	2561	-	Nitrogen fixation
Nitrogenase FeMo-cofactor carrier protein NifX	TAN_219_301	2630	3043	+	Nitrogen fixation
NifX-associated protein	TAN_219_301	3055	3516	+	Nitrogen fixation
NifT protein	TAN_219_337	1973	1779	-	Nitrogen fixation
NifZ protein	TAN_219_337	2244	1957	-	Nitrogen fixation
Homocitrate synthase (EC 2.3.3.14)	TAN_219_337	3364	2234	-	Nitrogen fixation
Nitrogenase-associated protein NifO	TAN_219_500	3165	2689	-	Nitrogen fixation
4Fe-4S ferredoxin, nitrogenase-associated	TAN_219_500	8686	8393	-	Nitrogen fixation
Molybdenum ABC transporter ModA (TC 3.A.1.8.1)	TAN_219_167	13959	14756	+	Molybdenum scavenging
Molybdenum transport system permease protein ModB (TC 3.A.1.8.1)	TAN_219_167	14890	16695	+	Molybdenum scavenging
gas vesicle protein	TAN_219_446	5006	4626	-	Buoyancy regulation
gas vesicle protein	TAN_219_446	5776	5018	-	Buoyancy regulation
gas vesicle protein	TAN_219_446	6304	5837	-	Buoyancy regulation
Gas vesicle protein gvpJ	TAN_219_446	7159	6446	-	Buoyancy regulation
Gas vesicle protein gvpN	TAN_219_446	8323	7394	-	Buoyancy regulation
gas vesicle structural protein	TAN_219_446	9155	8772	-	Buoyancy regulation
Gas vesicle protein	TAN_219_474	10695	10327	-	Buoyancy regulation
Glucose-1-phosphate adenylyltransferase (EC 2.7.7.27)	TAN_219_17	3912	5201	+	Buoyancy regulation
4-alpha-glucanotransferase (amylomaltase) (EC 2.4.1.25)	TAN_219_178	11506	13038	+	Buoyancy regulation
Glycogen branching enzyme, GH-57-type, archaeal (EC 2.4.1.18)	TAN_219_233	1570	50	-	Buoyancy regulation
Glycogen phosphorylase (EC 2.4.1.1)	TAN_219_27	7289	4725	-	Buoyancy regulation
1,4-alpha-glucan (glycogen) branching enzyme, GH-13-type (EC 2.4.1.18)	TAN_219_332	22064	19773	-	Buoyancy regulation
Glycogen synthase, ADP-glucose transglucosylase (EC 2.4.1.21)	TAN_219_495	5029	3614	-	Buoyancy regulation
ABC-type Co2+ transport system, permease component	TAN_219_27	3823	4293	+	CO2 fixation (Rubisco)/Calvin cycle
NADH dehydrogenase subunit 4, Involved in CO2 fixation	TAN_219_582	1832	3325	+	CO2 fixation (Rubisco)/Calvin cycle

Carboxysome protein CcmN	TAN_219_70	9081	9836	+	CO2 fixation (Rubisco)/Calvin cycle
Ribulose bisphosphate carboxylase large chain (EC 4.1.1.39)	TAN_219_100	19429	20859	+	CO2 fixation (Rubisco)/Calvin cycle
NADH dehydrogenase subunit 5, Involved in CO2 fixation	TAN_219_582	57	1781	+	CO2 fixation (Rubisco)/Calvin cycle
NADH dehydrogenase subunit 4, Involved in CO2 fixation	TAN_219_70	3602	2004	-	CO2 fixation (Rubisco)/Calvin cycle
Possible carbon dioxide concentrating mechanism protein CcmK	TAN_219_70	6526	6852	+	CO2 fixation (Rubisco)/Calvin cycle
Carboxysome protein CcmM	TAN_219_70	7323	8972	+	CO2 fixation (Rubisco)/Calvin cycle
Possible carbon dioxide concentrating mechanism protein CcmK	TAN_219_70	6101	6409	+	CO2 fixation (Rubisco)/Calvin cycle
NADH dehydrogenase subunit 5, Involved in CO2 fixation	TAN_219_70	5464	3608	-	CO2 fixation (Rubisco)/Calvin cycle
RuBisCO operon transcriptional regulator	TAN_219_195	387	1400	+	CO2 fixation (Rubisco)/Calvin cycle
Bicarbonate transport system permease protein	TAN_219_50	4267	5103	+	CO2 fixation (Rubisco)/Calvin cycle
RuBisCO operon transcriptional regulator	TAN_219_512	1909	1556	-	CO2 fixation (Rubisco)/Calvin cycle
Low-affinity CO2 hydration protein CphX	TAN_219_582	3554	4876	+	CO2 fixation (Rubisco)/Calvin cycle
Bicarbonate transport system permease protein	TAN_219_597	2580	3422	+	CO2 fixation (Rubisco)/Calvin cycle
Low-affinity CO2 hydration protein CphX	TAN_219_70	1944	814	-	CO2 fixation (Rubisco)/Calvin cycle
NADH dehydrogenase subunit 5, Involved in CO2 fixation	TAN_219_160	1046	2971	+	CO2 fixation (Rubisco)/Calvin cycle
High-affnity carbon uptake protein Hat/HatR	TAN_219_130	10394	9108	-	CO2 fixation (Rubisco)/Calvin cycle
Possible RuBisCo chaperonin RbcX	TAN_219_100	20961	21341	+	CO2 fixation (Rubisco)/Calvin cycle
Sensory subunit of low CO2-induced protein complex, putative	TAN_219_141	3179	2778	-	CO2 fixation (Rubisco)/Calvin cycle
putative sodium-dependent bicarbonate transporter	TAN_219_173	591	1565	+	CO2 fixation (Rubisco)/Calvin cycle
ribulose 1,5-bisphosphate carboxylase/oxygenase activase	TAN_219_392	6934	5708	-	CO2 fixation (Rubisco)/Calvin cycle
probable RuBisCo-expression protein CbbX	TAN_219_431	2303	1938	-	CO2 fixation (Rubisco)/Calvin cycle
Bicarbonate transporter, bicarbonate binding protein	TAN_219_50	2857	4233	+	CO2 fixation (Rubisco)/Calvin cycle
Carbon dioxide concentrating mechanism protein CcmO	TAN_219_554	13	426	+	CO2 fixation (Rubisco)/Calvin cycle
Carboxysome protein CcmL	TAN_219_70	6859	7164	+	CO2 fixation (Rubisco)/Calvin cycle
Ribulose bisphosphate carboxylase small chain (EC 4.1.1.39)	TAN_219_100	21564	21893	+	CO2 fixation (Rubisco)/Calvin cycle
Phosphoribulokinase (EC 2.7.1.19)	TAN_219_373	4272	5276	+	CO2 fixation (Rubisco)/Calvin cycle
Calvin cycle association	TAN_219_555	9095	9337	+	CO2 fixation (Rubisco)/Calvin cycle
Fructose-1,6-bisphosphatase, GlpX type (EC 3.1.3.11)	TAN_219_281	4660	5697	+	CO2 fixation (Rubisco)/Calvin cycle
Fructose-bisphosphate aldolase class II (EC 4.1.2.13)	TAN_219_450	1265	192	-	CO2 fixation (Rubisco)/Calvin cycle
NADPH-dependent glyceraldehyde-3-phosphate dehydrogenase (EC 1.2.1.13)	TAN_219_453	2303	1377	-	CO2 fixation (Rubisco)/Calvin cycle
Triosephosphate isomerase (EC 5.3.1.1)	TAN_219_323	1575	2315	+	CO2 fixation (Rubisco)/Calvin cycle
Fructose-1,6-bisphosphatase, type I (EC 3.1.3.11)	TAN_219_100	24815	25852	+	CO2 fixation (Rubisco)/Calvin cycle
Transketolase, N-terminal section (EC 2.2.1.1)	TAN_219_137	724	1503	+	CO2 fixation (Rubisco)/Calvin cycle
TPR domain protein, putative component of TonB system	TAN_219_100	22028	22858	+	Membrane transport
TPR domain protein, putative component of TonB system	TAN_219_171	6909	7130	+	Membrane transport
TPR domain protein, putative component of TonB system	TAN_219_256	9802	9059	-	Membrane transport
TPR domain protein, putative component of TonB system	TAN_219_265	2795	5128	+	Membrane transport
TPR domain protein, putative component of TonB system	TAN_219_370	1338	2180	+	Membrane transport

TPR domain protein, putative component of TonB system	TAN_219_394	7542	8843	+	Membrane transport
TPR domain protein, putative component of TonB system	TAN_219_420	3104	3643	+	Membrane transport
TPR domain protein, putative component of TonB system	TAN_219_481	4894	2753	-	Membrane transport
TPR domain protein, putative component of TonB system	TAN_219_5	3671	4543	+	Membrane transport
TPR domain protein, putative component of TonB system	TAN_219_598	8577	6571	-	Membrane transport
Cytochrome d ubiquinol oxidase subunit II (EC 1.10.3.-)	TAN_219_474	22814	23827	+	Oxygen respiration
Cytochrome c-type biogenesis protein Ccs1/ResB	TAN_219_589	7504	6581	-	Oxygen respiration
Cytochrome c-type biogenesis protein CcsA/ResC	TAN_219_420	2842	1781	-	Oxygen respiration
Cytochrome c-type biogenesis protein CcdA (DsbD analog)	TAN_219_72	488	1228	+	Oxygen respiration
Heme A synthase, cytochrome oxidase biogenesis protein Cox15-CtaA	TAN_219_570	1073	138	-	Oxygen respiration
Cytochrome d ubiquinol oxidase subunit I (EC 1.10.3.-)	TAN_219_474	21360	22802	+	Oxygen respiration

Supplementary Table 4. Metagenome assembled genome (MAG) of *Chlorobium* (TAN_426; Supplementary Table 2). Synthesis of the key genes and metabolic pathways presented in Fig. 3 and discussed in-text.

Gene	MAG contig	Start	Stop	Strand	Metabolic pathway
Homocitrate synthase (EC 2.3.3.14)	TAN_426_67	21042	22211	+	Nitrogen fixation
Nitrogenase (molybdenum-iron)-specific transcriptional regulator NifA	TAN_426_67	22217	23851	+	Nitrogen fixation
Nitrogenase (molybdenum-iron) reductase and maturation protein NifH	TAN_426_67	29012	29836	+	Nitrogen fixation
Nitrogen regulatory protein P-II, nitrogen-fixation associated, subunit A	TAN_426_67	29863	30216	+	Nitrogen fixation
Nitrogen regulatory protein P-II, nitrogen-fixation associated, subunit B	TAN_426_67	30216	30593	+	Nitrogen fixation
Nitrogenase (molybdenum-iron) alpha chain (EC 1.18.6.1)	TAN_426_67	30630	32264	+	Nitrogen fixation
Nitrogenase (molybdenum-iron) beta chain (EC 1.18.6.1)	TAN_426_67	32291	33670	+	Nitrogen fixation
Nitrogenase FeMo-cofactor scaffold and assembly protein NifE	TAN_426_67	33874	35271	+	Nitrogen fixation
Nitrogenase FeMo-cofactor scaffold and assembly protein NifN	TAN_426_67	35295	36644	+	Nitrogen fixation
Nitrogenase FeMo-cofactor synthesis NifB	TAN_426_67	36658	37932	+	Nitrogen fixation
Nitrogenase-associated protein NifO	TAN_426_17	82242	81802	-	Nitrogen fixation
Molybdenum ABC transporter ATP-binding protein ModC	TAN_426_30	123601	122705	-	Molybdenum scavenging
Molybdenum ABC transporter permease protein ModB	TAN_426_30	124266	123598	-	Molybdenum scavenging
Molybdenum ABC transporter, substrate-binding protein ModA	TAN_426_30	125384	124650	-	Molybdenum scavenging
Molybdenum transport system protein ModD	TAN_426_30	126169	125441	-	Molybdenum scavenging
Polyphosphate kinase (EC 2.7.4.1)	TAN_426_15	93747	91603	-	Buoyancy regulation
Exopolyphosphatase (EC 3.6.1.11)	TAN_426_67	113481	114413	+	Buoyancy regulation
Exopolyphosphatase (EC 3.6.1.11)	TAN_426_8	19524	17962	-	Buoyancy regulation
Glycogen phosphorylase (EC 2.4.1.1)	TAN_426_67	68917	71031	+	Buoyancy regulation
Glycogen synthase, ADP-glucose transglucosylase (EC 2.4.1.21)	TAN_426_73	34877	33408	-	Buoyancy regulation
Fumarate hydratase class I, aerobic (EC 4.2.1.2)	TAN_426_10	64114	62594	-	Reverse TCA
Fumarate hydratase class II (EC 4.2.1.2)	TAN_426_10	72873	71467	-	Reverse TCA
Succinyl-CoA ligase [ADP-forming] beta chain (EC 6.2.1.5)	TAN_426_16	29808	28633	-	Reverse TCA
Succinyl-CoA ligase [ADP-forming] alpha chain (EC 6.2.1.5)	TAN_426_47	1025	126	-	Reverse TCA
Isocitrate dehydrogenase [NADP] (EC 1.1.1.42)	TAN_426_8	21919	19694	-	Reverse TCA
Malate dehydrogenase (EC 1.1.1.37)	TAN_426_17	23347	22415	-	Reverse TCA
Aconitate hydratase 2 (EC 4.2.1.3)	TAN_426_23	5145	2575	-	Reverse TCA
Citrate synthase (si) (EC 2.3.3.1)	TAN_426_43	13619	12276	-	Reverse TCA
Aconitate hydratase (EC 4.2.1.3)	TAN_426_67	133805	131073	-	Reverse TCA
Dihydrolipoamide dehydrogenase (EC 1.8.1.4)	TAN_426_12	1719	3122	+	Reverse TCA
ATP citrate synthase, beta chain (EC 2.3.3.8)	TAN_426_15	63625	61793	-	Reverse TCA
ATP citrate synthase, alpha chain (EC 2.3.3.8)	TAN_426_15	64886	63690	-	Reverse TCA
2-oxoglutarate/2-oxoacid ferredoxin oxidoreductase, beta subunit (EC 1.2.7.-)	TAN_426_20	36379	35510	-	Reverse TCA
2-oxoglutarate/2-oxoacid ferredoxin oxidoreductase	TAN_426_20	38073	36376	-	Reverse TCA
Pyruvate:ferredoxin oxidoreductase, beta subunit (EC 1.2.7.1)	TAN_426_45	11599	12432	+	Reverse TCA

Pyruvate:ferredoxin oxidoreductase, beta subunit (EC 1.2.7.1)	TAN_426_45	12449	12676	+	Reverse TCA
Dissimilatory sulfite reductase, beta subunit (EC 1.8.99.5)	TAN_426_67	15186	14107	-	Sulfide oxidation
Dissimilatory sulfite reductase, alpha subunit (EC 1.8.99.5)	TAN_426_67	16532	15273	-	Sulfide oxidation
Sulfide:quinone oxidoreductase, Type III	TAN_426_20	15466	16692	+	Sulfide oxidation
putative TonB-dependent receptor	TAN_426_16	11760	12563	+	Membrane transport
TonB-dependent receptor	TAN_426_58	46253	48670	+	Membrane transport
Outer membrane receptor	TAN_426_3	1578	457	-	Membrane transport

Supplementary Table 5. Metagenome assembled genome (MAG) of *Aquabacterium* (TAN_398; Supplementary Table 2). Synthesis of the key genes and metabolic pathways presented in Fig. 3 and discussed in-text.

Gene	MAG contig	Start	Stop	Strand	Metabolic pathway
Nitrogenase (molybdenum-iron) alpha chain (EC 1.18.6.1)	TAN_398_226	1837	377	-	Nitrogen fixation
Nitrogenase (molybdenum-iron) reductase and maturation protein NifH	TAN_398_226	2869	1991	-	Nitrogen fixation
NifT protein	TAN_398_226	10376	10158	-	Nitrogen fixation
NifZ protein	TAN_398_226	12297	11983	-	Nitrogen fixation
NifZ protein	TAN_398_226	12659	12324	-	Nitrogen fixation
LRV (FeS)4 cluster domain protein clustered with nitrogenase cofactor synthesis	TAN_398_226	13101	12652	-	Nitrogen fixation
Homocitrate synthase (EC 2.3.3.14)	TAN_398_282	6307	7506	+	Nitrogen fixation
Nitrogenase stabilizing/protective protein NifW	TAN_398_314	437	811	+	Nitrogen fixation
Molybdenum transport ATP-binding protein ModC (TC 3.A.1.8.1)	TAN_398_333	6	908	+	Nitrogen fixation
Nitrogenase (molybdenum-iron) beta chain (EC 1.18.6.1)	TAN_398_387	1747	527	-	Nitrogen fixation
Nitrogenase FeMo-cofactor scaffold and assembly protein NifE	TAN_398_40	6042	7454	+	Nitrogen fixation
Nitrogenase FeMo-cofactor scaffold and assembly protein NifN	TAN_398_40	7467	8876	+	Nitrogen fixation
Nitrogenase FeMo-cofactor carrier protein NifX	TAN_398_40	8873	9292	+	Nitrogen fixation
NifX-associated protein	TAN_398_40	9285	9767	+	Nitrogen fixation
observed by proteomics Citation: Proteomics from VerBerkmoes et al. (2003) unpublished	TAN_398_40	9824	10018	+	Nitrogen fixation
4Fe-4S ferredoxin, nitrogenase-associated	TAN_398_40	10029	10340	+	Nitrogen fixation
Nitrogenase FeMo-cofactor synthesis FeS core scaffold and assembly protein NifB	TAN_398_58	2595	1012	-	Nitrogen fixation
Nitrogenase FeMo-cofactor synthesis molybdenum delivery protein NifQ	TAN_398_58	2880	3275	+	Nitrogen fixation
Nitrogenase (molybdenum-iron)-specific transcriptional regulator NifA	TAN_398_58	5021	3354	-	Nitrogen fixation
Molybdenum ABC transporter, periplasmic molybdenum-binding protein ModA (TC 3.A.1.8.1)	TAN_398_40	10527	11291	+	Molybdenum scavenging
Molybdenum transport system permease protein ModB (TC 3.A.1.8.1)	TAN_398_40	11304	11993	+	Molybdenum scavenging
Molybdenum transport ATP-binding protein ModC (TC 3.A.1.8.1)	TAN_398_40	11986	13092	+	Molybdenum scavenging
Molybdenum transport system protein ModD	TAN_398_40	13246	14091	+	Molybdenum scavenging
probable RuBisCo-expression protein CbbX	TAN_398_198	1448	2503	+	CO2 fixation (Rubisco)/Calvin cycle
Ribulose bisphosphate carboxylase large chain (EC 4.1.1.39)	TAN_398_198	165	983	+	CO2 fixation (Rubisco)/Calvin cycle
putative sodium-dependent bicarbonate transporter	TAN_398_199	7	360	+	CO2 fixation (Rubisco)/Calvin cycle
Rubisco activation protein CbbQ	TAN_398_212	2121	1297	-	CO2 fixation (Rubisco)/Calvin cycle
Sensory subunit of low CO2-induced protein complex, putative	TAN_398_216	485	994	+	CO2 fixation (Rubisco)/Calvin cycle
RuBisCO operon transcriptional regulator	TAN_398_294	17428	16472	-	CO2 fixation (Rubisco)/Calvin cycle
Ribulose bisphosphate carboxylase small chain (EC 4.1.1.39)	TAN_398_198	1002	1451	+	CO2 fixation (Rubisco)/Calvin cycle
Ribose 5-phosphate isomerase A (EC 5.3.1.6)	TAN_398_125	4649	3978	-	CO2 fixation (Rubisco)/Calvin cycle
Ribulose-phosphate 3-epimerase (EC 5.1.3.1)	TAN_398_51	12563	11850	-	CO2 fixation (Rubisco)/Calvin cycle

Fructose-1,6-bisphosphatase, type I (EC 3.1.3.11)	TAN_398_198	2494	3576	+	CO2 fixation (Rubisco)/Calvin cycle
Fructose-1,6-bisphosphatase, type I (EC 3.1.3.11)	TAN_398_344	7656	8669	+	CO2 fixation (Rubisco)/Calvin cycle
Phosphoribulokinase (EC 2.7.1.19)	TAN_398_257	112	588	+	CO2 fixation (Rubisco)/Calvin cycle
Phosphoglycerate kinase (EC 2.7.2.3)	TAN_398_150	4153	2960	-	CO2 fixation (Rubisco)/Calvin cycle
Fructose-bisphosphate aldolase class II (EC 4.1.2.13)	TAN_398_303	37078	36014	-	CO2 fixation (Rubisco)/Calvin cycle
Triosephosphate isomerase (EC 5.3.1.1)	TAN_398_153	1912	2658	+	CO2 fixation (Rubisco)/Calvin cycle
Transketolase (EC 2.2.1.1)	TAN_398_9	1962	559	-	CO2 fixation (Rubisco)/Calvin cycle
NAD-dependent glyceraldehyde-3-phosphate dehydrogenase (EC 1.2.1.12)	TAN_398_251	16298	15687	-	CO2 fixation (Rubisco)/Calvin cycle
TonB-dependent hemin , ferrichrome receptor	TAN_398_335	14673	12394	-	Membrane transport
Ferric siderophore transport system, periplasmic binding protein TonB	TAN_398_101	7003	6239	-	Membrane transport
Ferric siderophore transport system, periplasmic binding protein TonB	TAN_398_85	7605	8306	+	Membrane transport
Outer membrane receptor for ferric coprogen and ferric-rhodotorulic acid	TAN_398_124	11612	9462	-	Membrane transport
TonB-dependent receptor	TAN_398_3	38	1708	+	Membrane transport
TonB-dependent receptor; Outer membrane receptor for ferrienterochelin and colicins	TAN_398_384	8381	6411	-	Membrane transport
TolA protein	TAN_398_52	5010	4039	-	Membrane transport
TolA protein	TAN_398_59	3416	2454	-	Membrane transport
TonB domain protein	TAN_398_303	23385	24167	+	Membrane transport
TonB protein	TAN_398_313	555	1442	+	Membrane transport
TonB dependent receptor	TAN_398_265	15575	17710	+	Membrane transport
TRAP transporter solute receptor, TAXI family	TAN_398_129	34727	33336	-	Membrane transport
TRAP dicarboxylate transporter, DctM subunit, unknown substrate 3	TAN_398_449	5162	4800	-	Membrane transport
TRAP-type C4-dicarboxylate transport system, large permease component	TAN_398_399	4828	3548	-	Membrane transport
TRAP dicarboxylate transporter, DctM subunit, unknown substrate 6	TAN_398_308	1827	3383	+	Membrane transport
TRAP dicarboxylate transporter, DctQ subunit, unknown substrate 6	TAN_398_308	1297	1830	+	Membrane transport
TRAP-type C4-dicarboxylate transport system, large permease component	TAN_398_286	3929	6082	+	Membrane transport
TRAP-type C4-dicarboxylate transport system, periplasmic component	TAN_398_100	2641	3666	+	Membrane transport
TRAP-type C4-dicarboxylate transport system, small permease component	TAN_398_399	5097	4840	-	Membrane transport
TRAP transporter solute receptor, unknown substrate 6	TAN_398_308	5356	4274	-	Membrane transport
ABC transporter, transmembrane region:ABC transporter:Peptidase C39, bacteriocin processing	TAN_398_1	6041	4287	-	Membrane transport
ABC transporter ATP-binding protein	TAN_398_111	9788	11341	+	Membrane transport
Nucleoside ABC transporter, periplasmic nucleoside-binding protein	TAN_398_117	4513	5367	+	Membrane transport
Maltose/maltodextrin ABC transporter, permease protein MalG	TAN_398_123	1399	542	-	Membrane transport
ABC-type sugar transport system, permease component	TAN_398_123	2305	1412	-	Membrane transport
ABC-type sugar transport system, periplasmic component	TAN_398_123	3704	2442	-	Membrane transport
Urea ABC transporter, urea binding protein	TAN_398_363	701	1954	+	Membrane transport
ABC-type amino acid transport, signal transduction systems, periplasmic component/domain	TAN_398_425	504	3356	+	Membrane transport

ABC-type nitrate/sulfonate/bicarbonate transport system, ATPase component	TAN_398_431	2803	3585	+	Membrane transport
Amino acid ABC transporter, permease protein	TAN_398_58	8676	9461	+	Membrane transport
Branched-chain amino acid transport system permease protein LivM (TC 3.A.1.4.1)	TAN_398_265	2175	1072	-	Membrane transport
Branched-chain amino acid transport ATP-binding protein LivF (TC 3.A.1.4.1)	TAN_398_358	9732	10427	+	Membrane transport
Branched-chain amino acid transport ATP-binding protein LivF (TC 3.A.1.4.1)	TAN_398_394	12724	13440	+	Membrane transport
Branched-chain amino acid transport system permease protein LivM (TC 3.A.1.4.1)	TAN_398_83	1541	2527	+	Membrane transport
Branched-chain amino acid transport ATP-binding protein LivF (TC 3.A.1.4.1)	TAN_398_99	5611	6351	+	Membrane transport
Cyanate ABC transporter, ATP-binding protein	TAN_398_51	22031	21144	-	Membrane transport
Ribose ABC transport system, permease protein RbsC (TC 3.A.1.2.1)	TAN_398_181	2696	1788	-	Membrane transport
Ribose ABC transport system, permease protein RbsC (TC 3.A.1.2.1)	TAN_398_181	3850	2708	-	Membrane transport
Fructose ABC transporter, ATP-binding component FrcA	TAN_398_416	6271	5465	-	Membrane transport
Ferric iron ABC transporter, iron-binding protein	TAN_398_76	5026	6045	+	Membrane transport
Lipopolysaccharide ABC transporter, ATP-binding protein LptB	TAN_398_423	5413	4625	-	Membrane transport
Lipoprotein releasing system ATP-binding protein LolD	TAN_398_83	13206	13922	+	Membrane transport
Methionine ABC transporter ATP-binding protein	TAN_398_323	27213	26182	-	Membrane transport
Dipeptide transport ATP-binding protein DppF (TC 3.A.1.5.2)	TAN_398_158	6091	5282	-	Membrane transport
Dipeptide transport system permease protein DppC (TC 3.A.1.5.2)	TAN_398_187	68939	69757	+	Membrane transport
Dipeptide-binding ABC transporter, periplasmic substrate-binding component (TC 3.A.1.5.2)	TAN_398_211	5429	7678	+	Membrane transport
Oligopeptide transport system permease protein OppB (TC 3.A.1.5.1)	TAN_398_211	7678	8661	+	Membrane transport
Oligopeptide ABC transporter, periplasmic oligopeptide-binding protein OppA (TC 3.A.1.5.1)	TAN_398_362	37575	36238	-	Membrane transport
Oligopeptidase A (EC 3.4.24.70)	TAN_398_125	1192	3243	+	Degradation
3-isopropylmalate dehydrogenase (EC 1.1.1.85)	TAN_398_25	176	1258	+	Degradation
3-isopropylmalate dehydratase large subunit (EC 4.2.1.33)	TAN_398_389	1648	3057	+	Degradation
Muconolactone isomerase (EC 5.3.3.4)	TAN_398_76	50225	49935	-	Degradation
3-oxoadipate CoA-transferase subunit A (EC 2.8.3.6)	TAN_398_121	4705	4307	-	Degradation
Catechol 1,2-dioxygenase (EC 1.13.11.1)	TAN_398_76	49879	48956	-	Degradation
Alpha-ketoglutarate-dependent taurine dioxygenase (EC 1.14.11.17)	TAN_398_323	27820	28701	+	Degradation
Nitrilotriacetate monooxygenase component B (EC 1.14.13.-)	TAN_398_187	19129	18506	-	Degradation
Nitrilotriacetate monooxygenase component B (EC 1.14.13.-)	TAN_398_362	46706	46185	-	Degradation
Catechol 1,2-dioxygenase (EC 1.13.11.1)	TAN_398_127	8818	9732	+	Degradation
Benzoate 1,2-dioxygenase alpha subunit (EC 1.14.12.10)	TAN_398_127	9754	11124	+	Degradation
3-phenylpropionate dioxygenase, beta subunit (EC 1.14.12.19)	TAN_398_132	22845	23384	+	Degradation
ferredoxin subunit of phenylpropionate dioxygenase	TAN_398_132	21076	21420	+	Degradation

benzoate dioxygenase, ferredoxin reductase component	TAN_398_127	11683	12690	+	Degradation
Large subunit naph/bph dioxygenase	TAN_398_132	21439	22845	+	Degradation
Dioxygenases related to 2-nitropropane dioxygenase	TAN_398_132	24239	25276	+	Degradation
Cyclohexanone monooxygenase (EC 1.14.13.22)	TAN_398_151	13370	11793	-	Degradation
Cyclohexanone monooxygenase (EC 1.14.13.22)	TAN_398_163	735	2240	+	Degradation
Cyclohexanone monooxygenase (EC 1.14.13.22)	TAN_398_184	11774	13294	+	Degradation
Cyclohexanone monooxygenase (EC 1.14.13.22)	TAN_398_187	46129	44444	-	Degradation
Dioxygenases related to 2-nitropropane dioxygenase	TAN_398_222	6171	5194	-	Degradation
Phenylpropionate dioxygenase	TAN_398_227	3730	2570	-	Degradation
2-nitropropane dioxygenase (EC 1.13.11.32)	TAN_398_364	3519	4760	+	Degradation
Alkane-1 monooxygenase (EC 1.14.15.3)	TAN_398_57	10238	9042	-	Degradation
Flagellar basal-body rod modification protein FlgD	TAN_398_36	6608	5952	-	Motility
Chemotaxis regulator CheY	TAN_398_53	8231	8548	+	Motility
Flagellar biosynthesis protein FlhA	TAN_398_89	15362	13275	-	Motility
Flagellar motor rotation protein MotA	TAN_398_72	3456	2599	-	Motility
Flagellar biosynthesis protein FliR	TAN_398_373	2335	3099	+	Motility
Flagellar transcriptional activator FlhC	TAN_398_275	4613	4077	-	Motility
Flagellar motor switch protein FliG	TAN_398_350	1516	2514	+	Motility
Flagellar biosynthesis protein FliP	TAN_398_373	1264	2043	+	Motility
Flagellar hook-associated protein FliD	TAN_398_395	5197	6702	+	Motility
Flagellar hook-associated protein FliD	TAN_398_395	6981	8105	+	Motility
Flagellar protein FliJ	TAN_398_404	1789	2268	+	Motility
Flagellar hook-associated protein FlgL	TAN_398_86	1485	2423	+	Motility
Flagellar biosynthesis protein FlhB	TAN_398_89	16552	15374	-	Motility
Flagellar motor switch protein FliM	TAN_398_373	112	378	+	Motility
Chemotaxis regulator CheY	TAN_398_72	1521	1141	-	Motility
Flagellar P-ring protein FlgI	TAN_398_36	2337	1207	-	Motility
Flagellar biosynthesis protein FliO	TAN_398_373	887	1246	+	Motility
Flagellar biosynthesis protein FliS	TAN_398_395	8274	8702	+	Motility
Flagellar transcriptional activator FlhC	TAN_398_72	4274	3738	-	Motility
Flagellar basal-body rod protein FlgC	TAN_398_36	7037	6627	-	Motility
Flagellar biosynthesis protein FlhF	TAN_398_89	13278	11569	-	Motility
RNA polymerase sigma factor for flagellar operon	TAN_398_89	10680	9967	-	Motility
Flagellar basal-body rod protein FlgB	TAN_398_36	7506	7066	-	Motility
Flagellin protein FlaA	TAN_398_395	2938	3873	+	Motility
Flagellar L-ring protein FlgH	TAN_398_36	3045	2380	-	Motility
Flagellar L-ring protein FlgH	TAN_398_127	4037	4771	+	Motility
Flagellar synthesis regulator FleN	TAN_398_253	29809	29000	-	Motility
Flagellar synthesis regulator FleN	TAN_398_89	11542	10697	-	Motility

Flagellar transcriptional activator FlhD	TAN_398_275	4998	4675	-	Motility
Flagellar transcriptional activator FlhD	TAN_398_72	4641	4318	-	Motility
Signal transduction histidine kinase CheA (EC 2.7.3.-)	TAN_398_77	7	972	+	Motility
Signal transduction histidine kinase CheA (EC 2.7.3.-)	TAN_398_149	27	452	+	Motility
Signal transduction histidine kinase CheA (EC 2.7.3.-)	TAN_398_53	8849	11104	+	Motility
Chemotaxis regulator CheY	TAN_398_316	2902	557	-	Motility
Chemotaxis regulator CheY	TAN_398_364	11478	11840	+	Motility
Predicted signal transduction protein	TAN_398_86	2445	3653	+	Motility
Flagellar motor rotation protein MotB	TAN_398_72	2556	1591	-	Motility
Flagellum-specific ATP synthase FliI	TAN_398_404	324	1742	+	Motility
Flagellar motor switch protein FliN	TAN_398_373	371	862	+	Motility
Flagellar biosynthesis protein FliL	TAN_398_186	411	1043	+	Motility
Flagellar basal-body P-ring formation protein FlgA	TAN_398_220	5335	4715	-	Motility
Flagellar M-ring protein FliF	TAN_398_350	7	1497	+	Motility
Flagellar hook protein FlgE	TAN_398_36	5917	4631	-	Motility
Flagellar assembly protein FliH	TAN_398_404	22	315	+	Motility
Flagellar hook-length control protein FliK	TAN_398_422	17	871	+	Motility
Flagellar hook-associated protein FlgK	TAN_398_86	60	1466	+	Motility
Flagellar hook-basal body complex protein FliE	TAN_398_243	1886	1527	-	Motility
Nitrite transporter from formate/nitrite family	TAN_398_187	73893	73081	-	Nitrate reduction
Nitrate/nitrite response regulator protein	TAN_398_201	5915	5247	-	Nitrate reduction
Nitrate ABC transporter, ATP-binding protein	TAN_398_270	4473	5273	+	Nitrate reduction
Nitrate reductase cytochrome c550-type subunit	TAN_398_91	3057	3521	+	Nitrate reduction
Nitrite reductase [NAD(P)H] large subunit (EC 1.7.1.4)	TAN_398_209	9269	10558	+	Nitrate reduction
Nitrate/nitrite transporter	TAN_398_268	2195	741	-	Nitrate reduction
Nitrate/nitrite transporter	TAN_398_268	3506	2220	-	Nitrate reduction
Nitrate/nitrite sensor protein (EC 2.7.3.-)	TAN_398_268	5466	7436	+	Nitrate reduction
Periplasmic nitrate reductase precursor (EC 1.7.99.4)	TAN_398_91	534	3038	+	Nitrate reduction
periplasmic diheme c-type cytochrome, NapB	TAN_398_91	220	537	+	Nitrate reduction
Respiratory nitrate reductase alpha chain (EC 1.7.99.4)	TAN_398_268	710	117	-	Nitrate reduction
Respiratory nitrate reductase alpha chain (EC 1.7.99.4)	TAN_398_349	3575	180	-	Nitrate reduction
Nitric oxide-dependent regulator DnrN or NorA	TAN_398_280	1894	1241	-	Nitrate reduction
Nitric oxide-dependent regulator DnrN or NorA	TAN_398_335	19215	18757	-	Nitrate reduction
Nitrate/nitrite sensor protein (EC 2.7.3.-)	TAN_398_201	7366	5915	-	Nitrate reduction
Nitrate/nitrite transporter	TAN_398_209	6065	7348	+	Nitrate reduction
Nitrate/nitrite sensor protein (EC 2.7.3.-)	TAN_398_364	10660	9506	-	Nitrate reduction
Response regulator NasT	TAN_398_187	75507	74182	-	Nitrate reduction
Nitrite reductase [NAD(P)H] small subunit (EC 1.7.1.4)	TAN_398_187	76152	75611	-	Nitrate reduction
Assimilatory nitrate reductase large subunit (EC:1.7.99.4)	TAN_398_209	10668	13508	+	Nitrate reduction

Nitrate ABC transporter, nitrate-binding protein	TAN_398_270	1959	3269	+	Nitrate reduction
Respiratory nitrate reductase delta chain (EC 1.7.99.4)	TAN_398_388	693	1541	+	Nitrate reduction
Respiratory nitrate reductase gamma chain (EC 1.7.99.4)	TAN_398_388	1538	2239	+	Nitrate reduction
Cytochrome c-type protein NapC	TAN_398_91	3539	4120	+	Oxygen respiration
Cytochrome c-type biogenesis protein Ccs1/ResB	TAN_398_303	29406	27124	-	Oxygen respiration
Cytochrome c-type biogenesis protein CcmC, putative heme lyase for CcmE	TAN_398_91	5420	6160	+	Oxygen respiration
Cytochrome oxidase biogenesis protein Sco1/SenC/PrrC	TAN_398_55	1387	2022	+	Oxygen respiration
Cytochrome c oxidase polypeptide I (EC 1.9.3.1)	TAN_398_18	3935	2781	-	Oxygen respiration
Type cbb3 cytochrome oxidase biogenesis protein CcoG, involved in Cu oxidation	TAN_398_12	2370	895	-	Oxygen respiration
Cytochrome c oxidase polypeptide II (EC 1.9.3.1)	TAN_398_151	20043	19180	-	Oxygen respiration
Transport ATP-binding protein CydC	TAN_398_76	64969	63266	-	Oxygen respiration
Transport ATP-binding protein CydD	TAN_398_76	66716	64983	-	Oxygen respiration
Ubiquinol-cytochrome C reductase iron-sulfur subunit (EC 1.10.2.2)	TAN_398_340	2342	2965	+	Oxygen respiration
Ubiquinol--cytochrome c reductase, cytochrome B subunit (EC 1.10.2.2)	TAN_398_340	2980	4380	+	Oxygen respiration
ABC transporter involved in cytochrome c biogenesis, ATPase component CcmA	TAN_398_91	4120	4755	+	Oxygen respiration
Cytochrome c-type biogenesis protein CcmE, heme chaperone	TAN_398_91	6366	6770	+	Oxygen respiration
Cytochrome d ubiquinol oxidase subunit II (EC 1.10.3.-)	TAN_398_384	1718	2725	+	Oxygen respiration
Cytochrome d ubiquinol oxidase subunit II (EC 1.10.3.-)	TAN_398_76	70818	69682	-	Oxygen respiration
Cytochrome c-type biogenesis protein ResA	TAN_398_178	8284	9301	+	Oxygen respiration
Cytochrome c-type biogenesis protein ResA	TAN_398_92	12839	12318	-	Oxygen respiration
Cytochrome c oxidase polypeptide III (EC 1.9.3.1)	TAN_398_18	1637	819	-	Oxygen respiration
Cytochrome c oxidase polypeptide II (EC 1.9.3.1)	TAN_398_305	11244	12326	+	Oxygen respiration
Cytochrome c oxidase polypeptide I (EC 1.9.3.1)	TAN_398_151	19183	16634	-	Oxygen respiration
Cytochrome d ubiquinol oxidase subunit I (EC 1.10.3.-)	TAN_398_384	319	1716	+	Oxygen respiration
Cytochrome d ubiquinol oxidase subunit I (EC 1.10.3.-)	TAN_398_76	71697	70840	-	Oxygen respiration
Cytochrome c oxidase polypeptide I (EC 1.9.3.1)	TAN_398_63	8208	6601	-	Oxygen respiration
Type cbb3 cytochrome oxidase biogenesis protein CcoI	TAN_398_156	3303	1006	-	Oxygen respiration

Supplementary Table 6. Metagenome assembled genome (MAG) of *Pseudomonas* (TAN_78; Supplementary Table 2). Synthesis of the key genes and metabolic pathways presented in Fig. 3 and discussed in-text.

Gene	MAG contig	Start	Stop	Strand	Metabolic pathway
Nitrogenase FeMo-cofactor synthesis molybdenum delivery protein NifQ	TAN_78_220	1641	1084	-	Nitrogen fixation
Nitrogenase-associated protein NifO	TAN_78_220	2227	1638	-	Nitrogen fixation
Nitrogenase FeMo-cofactor synthesis FeS core scaffold and assembly protein NifB	TAN_78_220	3902	2385	-	Nitrogen fixation
Nitrogenase (molybdenum-iron)-specific transcriptional regulator NifA	TAN_78_220	10441	8876	-	Nitrogen fixation
Nitrogenase cofactor carrier protein NafY	TAN_78_220	17881	18612	+	Nitrogen fixation
nitrogen fixation-related protein	TAN_78_220	18609	19007	+	Nitrogen fixation
hypothetical protein	TAN_78_220	19932	19363	-	Nitrogen fixation
hypothetical protein	TAN_78_220	20805	19954	-	Nitrogen fixation
Nitrogenase (molybdenum-iron) reductase and maturation protein NifH	TAN_78_220	21222	22103	+	Nitrogen fixation
Nitrogenase (molybdenum-iron) alpha chain (EC 1.18.6.1)	TAN_78_220	22215	23696	+	Nitrogen fixation
Nitrogenase (molybdenum-iron) beta chain (EC 1.18.6.1)	TAN_78_220	23799	25370	+	Nitrogen fixation
NifT protein	TAN_78_220	25462	25683	+	Nitrogen fixation
NifY protein	TAN_78_220	25687	26421	+	Nitrogen fixation
hypothetical protein	TAN_78_220	26418	26687	+	Nitrogen fixation
LRV (FeS)4 cluster domain protein clustered with nitrogenase cofactor synthesis	TAN_78_220	26697	27428	+	Nitrogen fixation
Nitrogenase FeMo-cofactor scaffold and assembly protein NifE	TAN_78_220	27840	29261	+	Nitrogen fixation
Nitrogenase FeMo-cofactor carrier protein NifX	TAN_78_220	30392	31131	+	Nitrogen fixation
NifX-associated protein	TAN_78_220	31160	31639	+	Nitrogen fixation
4Fe-4S ferredoxin, nitrogenase-associated	TAN_78_220	31946	32242	+	Nitrogen fixation
Nitrogenase stabilizing/protective protein NifW	TAN_78_220	43856	44203	+	Nitrogen fixation
NifZ protein	TAN_78_220	44223	44693	+	Nitrogen fixation
NifM protein	TAN_78_220	44683	45561	+	Nitrogen fixation
Molybdenum transport ATP-binding protein ModC (TC 3.A.1.8.1)	TAN_78_220	33258	34192	+	Molybdenum scavenging
Molybdenum transport ATP-binding protein ModC (TC 3.A.1.8.1)	TAN_78_220	36178	35081	-	Molybdenum scavenging
Molybdenum transport system permease protein ModB (TC 3.A.1.8.1)	TAN_78_220	36871	36191	-	Molybdenum scavenging
Molybdenum ABC transporter, periplasmic molybdenum-binding protein ModA (TC 3.A.1.8.1)	TAN_78_220	37630	36878	-	Molybdenum scavenging
TonB-dependent receptor, in a cluster with 3-phytase	TAN_78_290	89	2572	+	Membrane transport
Periplasmic protein TonB, links inner and outer membranes	TAN_78_55	1470	2276	+	Membrane transport
tolB protein precursor, periplasmic protein involved in the tonb-independent uptake of group A colicins	TAN_78_165	1613	312	-	Membrane transport
Ferric siderophore transport system, periplasmic binding protein TonB	TAN_78_273	10230	9517	-	Membrane transport
Ferric siderophore transport system, periplasmic binding protein TonB	TAN_78_290	5820	6608	+	Membrane transport
TonB-dependent receptor; Outer membrane receptor for ferrienterochelin and colicins	TAN_78_241	47400	49622	+	Membrane transport

Outer membrane lipoprotein omp16 precursor	TAN_78_257	3798	4580	+	Membrane transport
TPR domain protein, putative component of TonB system	TAN_78_273	9515	8424	-	Membrane transport
TRAP-type C4-dicarboxylate transport system, periplasmic component	TAN_78_161	15304	14306	-	Membrane transport
TRAP-type C4-dicarboxylate transport system, periplasmic component	TAN_78_264	1494	2486	+	Membrane transport
TRAP-type C4-dicarboxylate transport system, periplasmic component	TAN_78_267	31094	30102	-	Membrane transport
TRAP-type C4-dicarboxylate transport system, periplasmic component	TAN_78_52	5410	6405	+	Membrane transport
Phosphonate ABC transporter phosphate-binding periplasmic component (TC 3.A.1.9.1)	TAN_78_271	5752	6309	+	Membrane transport
Phosphonate ABC transporter phosphate-binding periplasmic component (TC 3.A.1.9.1)	TAN_78_313	496	1428	+	Membrane transport
Phosphonate ABC transporter phosphate-binding periplasmic component (TC 3.A.1.9.1)	TAN_78_76	11733	11455	-	Membrane transport
Phosphonate ABC transporter phosphate-binding periplasmic component (TC 3.A.1.9.1)	TAN_78_83	20219	19635	-	Membrane transport
Phosphonate ABC transporter phosphate-binding periplasmic component (TC 3.A.1.9.1)	TAN_78_84	9288	9665	+	Membrane transport
Phosphonate ABC transporter phosphate-binding periplasmic component (TC 3.A.1.9.1)	TAN_78_87	1822	1319	-	Membrane transport
TRAP-type transport system, periplasmic component, predicted N-acetylneuraminate-binding protein	TAN_78_294	7157	6141	-	Membrane transport
TRAP transporter, 4TM/12TM fusion protein, unknown substrate 1	TAN_78_213	3122	1098	-	Membrane transport
TRAP-type C4-dicarboxylate transport system, periplasmic component	TAN_78_122	3262	2324	-	Membrane transport
TRAP-type C4-dicarboxylate transport system, periplasmic component	TAN_78_122	4640	3690	-	Membrane transport
TRAP dicarboxylate transporter, DctQ subunit, unknown substrate 3	TAN_78_34	15783	15256	-	Membrane transport
TRAP dicarboxylate transporter, DctM subunit, unknown substrate 6	TAN_78_110	1935	3305	+	Membrane transport
TRAP dicarboxylate transporter, DctM subunit, unknown substrate 6	TAN_78_8	10554	11930	+	Membrane transport
TRAP transporter solute receptor, unknown substrate 3	TAN_78_34	16885	15830	-	Membrane transport
TRAP dicarboxylate transporter, DctQ subunit, unknown substrate 6	TAN_78_110	1316	1918	+	Membrane transport
TRAP dicarboxylate transporter, DctQ subunit, unknown substrate 6	TAN_78_8	10016	10561	+	Membrane transport
TRAP-type C4-dicarboxylate transport system, large permease component	TAN_78_294	8496	7219	-	Membrane transport
TRAP-type C4-dicarboxylate transport system, large permease component	TAN_78_52	7075	8577	+	Membrane transport
TRAP transporter solute receptor, TAXI family precursor	TAN_78_28	2295	1330	-	Membrane transport
TRAP transporter solute receptor, TAXI family precursor	TAN_78_294	13610	14587	+	Membrane transport
TRAP transporter solute receptor, unknown substrate 6	TAN_78_110	155	1240	+	Membrane transport
TRAP transporter solute receptor, unknown substrate 6	TAN_78_306	4644	3817	-	Membrane transport
TRAP transporter solute receptor, unknown substrate 6	TAN_78_8	9720	8614	-	Membrane transport
Amino acid ABC transporter, permease protein	TAN_78_285	1668	979	-	Membrane transport
TRAP transporter, 4TM/12TM fusion protein, unknown substrate 1	TAN_78_213	3122	1098	-	Membrane transport
Phosphonate ABC transporter ATP-binding protein (TC 3.A.1.9.1)	TAN_78_47	12498	11644	-	Membrane transport
Branched-chain amino acid transport ATP-binding protein LivF (TC 3.A.1.4.1)	TAN_78_137	15057	14353	-	Membrane transport
Branched-chain amino acid transport ATP-binding protein LivF (TC 3.A.1.4.1)	TAN_78_259	34049	33333	-	Membrane transport

High-affinity branched-chain amino acid transport system permease protein LivH (TC 3.A.1.4.1)	TAN_78_47	8131	7208	-	Membrane transport
Alkanesulfonates ABC transporter ATP-binding protein / Sulfonate ABC transporter, ATP-binding subunit SsuB	TAN_78_80	1612	821	-	Membrane transport
Lipopolysaccharide ABC transporter, ATP-binding protein LptB	TAN_78_241	16255	16980	+	Membrane transport
Lipoprotein releasing system ATP-binding protein LolD	TAN_78_102	7083	7796	+	Membrane transport
Lipoprotein releasing system ATP-binding protein LolD	TAN_78_293	16280	16978	+	Membrane transport
Maltose/maltodextrin ABC transporter, permease protein MalF	TAN_78_263	3605	5170	+	Membrane transport
Methionine ABC transporter ATP-binding protein	TAN_78_123	4190	3183	-	Membrane transport
Glucose ABC transporter, ATP-binding subunit (EC 3.6.3.-)	TAN_78_204	34814	33642	-	Membrane transport
Urea ABC transporter, permease protein UrtC	TAN_78_114	10903	11982	+	Membrane transport
Urea carboxylase-related ABC transporter, ATPase protein	TAN_78_12	1706	2545	+	Membrane transport
Urea carboxylase-related aminomethyltransferase (EC 2.1.2.10)	TAN_78_160	4913	5641	+	Membrane transport
Oligopeptide transport system permease protein OppB (TC 3.A.1.5.1)	TAN_78_147	2440	779	-	Membrane transport
Oligopeptide transport system permease protein OppB (TC 3.A.1.5.1)	TAN_78_147	4536	3463	-	Membrane transport
Oligopeptide ABC transporter, periplasmic oligopeptide-binding protein OppA (TC 3.A.1.5.1)	TAN_78_147	8290	6461	-	Membrane transport
Branched-chain amino acid transport system permease protein LivM (TC 3.A.1.4.1)	TAN_78_137	13466	12480	-	Membrane transport
Branched-chain amino acid transport system permease protein LivM (TC 3.A.1.4.1)	TAN_78_259	31079	30072	-	Membrane transport
Branched-chain amino acid transport ATP-binding protein LivF (TC 3.A.1.4.1)	TAN_78_47	5188	4487	-	Membrane transport
Muconolactone isomerase (EC 5.3.3.4)	TAN_78_34	26746	27036	+	Membrane transport
Beta-ketoadipate enol-lactone hydrolase (EC 3.1.1.24)	TAN_78_125	29093	28305	-	Membrane transport
Protocatechuate 3,4-dioxygenase beta chain (EC 1.13.11.3)	TAN_78_125	37991	37272	-	Membrane transport
Branched-chain amino acid transport ATP-binding protein LivG (TC 3.A.1.4.1)	TAN_78_137	15793	15050	-	Membrane transport
Branched-chain amino acid transport ATP-binding protein LivG (TC 3.A.1.4.1)	TAN_78_259	34807	34046	-	Membrane transport
Branched-chain amino acid transport system permease protein LivM (TC 3.A.1.4.1)	TAN_78_47	7211	5955	-	Membrane transport
Oligopeptidase A (EC 3.4.24.70)	TAN_78_18	32373	30322	-	Degradation
Protocatechuate 3,4-dioxygenase alpha chain (EC 1.13.11.3)	TAN_78_125	37259	36654	-	Degradation
4-hydroxyphenylpyruvate dioxygenase (EC 1.13.11.27)	TAN_78_180	115	1617	+	Degradation
Benzoate 1,2-dioxygenase alpha subunit (EC 1.14.12.10)	TAN_78_34	20405	21766	+	Degradation
biphenyl-2,3-diol 1,2-dioxygenase III-related protein	TAN_78_70	2594	2190	-	Degradation
Homogentisate 1,2-dioxygenase (EC 1.13.11.5)	TAN_78_69	12364	13503	+	Degradation
1,2-dihydroxy-3-keto-5-methylthiopentene dioxygenase (EC 1.13.11.54)	TAN_78_204	27779	27234	-	Degradation
2-nitropropane dioxygenase, NPD	TAN_78_227	10050	10472	+	Degradation
benzoate dioxygenase, ferredoxin reductase component	TAN_78_34	22268	23278	+	Degradation
Phytanoyl-CoA dioxygenase	TAN_78_42	1403	642	-	Degradation
Catechol 1,2-dioxygenase (EC 1.13.11.1)	TAN_78_34	27108	28046	+	Degradation

Flagellar motor rotation protein MotA	TAN_78_102	1995	2804	+	Motility
Flagellar basal-body rod modification protein FlgD	TAN_78_310	3027	2344	-	Motility
Flagellar basal-body rod modification protein FlgD	TAN_78_316	9842	9177	-	Motility
Chemotaxis regulator - transmits chemoreceptor signals to flagelllar motor components CheY	TAN_78_43	21601	21236	-	Motility
Flagellar motor switch protein FliM	TAN_78_201	2169	1198	-	Motility
Flagellar motor rotation protein MotA	TAN_78_119	1124	273	-	Motility
Flagellar regulatory protein FleQ	TAN_78_102	24442	23048	-	Motility
Flagellar regulatory protein FleQ	TAN_78_102	27233	25767	-	Motility
Flagellar M-ring protein FliF	TAN_78_201	12382	10598	-	Motility
Flagellar biosynthesis protein FliP	TAN_78_30	27114	26515	-	Motility
Flagellar motor rotation protein MotA	TAN_78_30	13736	12996	-	Motility
Flagellar biosynthesis protein FlhB	TAN_78_30	25418	24282	-	Motility
Flagellar biosynthesis protein FlhA	TAN_78_30	23502	21382	-	Motility
Signal transduction histidine kinase CheA (EC 2.7.3.-)	TAN_78_30	17133	14905	-	Motility
Flagellar sensor histidine kinase FleS	TAN_78_102	25634	24447	-	Motility
Flagellar biosynthesis protein FliQ	TAN_78_201	589	254	-	Motility
Flagellar basal-body rod protein FlgF	TAN_78_225	1196	1936	+	Motility
Flagellar P-ring protein FlgI	TAN_78_225	3549	4649	+	Motility
Flagellar basal-body rod protein FlgC	TAN_78_310	3490	3047	-	Motility
Flagellar biosynthesis protein FlhF	TAN_78_30	21366	20074	-	Motility
RNA polymerase sigma factor for flagellar operon	TAN_78_30	19137	18412	-	Motility
Flagellar biosynthesis protein FliQ	TAN_78_30	26502	26233	-	Motility
Flagellar basal-body P-ring formation protein FlgA	TAN_78_310	6326	6718	+	Motility
Flagellar biosynthesis protein FlgN	TAN_78_310	7195	7665	+	Motility
Flagellar motor switch protein FliG	TAN_78_201	10605	9589	-	Motility
Chemotaxis regulator - transmits chemoreceptor signals to flagelllar motor components CheY	TAN_78_30	18349	17963	-	Motility
Flagellar basal-body rod protein FlgG	TAN_78_225	1972	2757	+	Motility
Flagellar basal-body rod protein FlgF	TAN_78_316	7926	7198	-	Motility
Flagellar L-ring protein FlgH	TAN_78_225	2839	3534	+	Motility
Flagellar L-ring protein FlgH	TAN_78_316	6398	5730	-	Motility
Chemotaxis protein CheV (EC 2.7.3.-)	TAN_78_190	3903	2971	-	Motility
Chemotaxis protein CheV (EC 2.7.3.-)	TAN_78_310	5891	4959	-	Motility
Flagellar biosynthesis protein FliL	TAN_78_201	2716	2183	-	Motility
Flagellar hook-associated protein FlgK	TAN_78_225	5840	7855	+	Motility
Flagellar hook-associated protein FlgK	TAN_78_316	3499	2120	-	Motility
Flagellar biosynthesis protein FliL	TAN_78_88	28934	28548	-	Motility
Signal transduction histidine kinase CheA (EC 2.7.3.-)	TAN_78_43	20903	18657	-	Motility

Flagellar motor rotation protein MotB	TAN_78_102	2807	3541	+	Motility
Flagellar motor rotation protein MotB	TAN_78_138	106	846	+	Motility
Flagellar motor rotation protein MotB	TAN_78_30	12990	12154	-	Motility
Signal transduction histidine kinase CheA (EC 2.7.3.-)	TAN_78_133	9630	6058	-	Motility
Flagellar hook protein FlgE	TAN_78_225	728	997	+	Motility
Flagellar hook protein FlgE	TAN_78_310	2313	844	-	Motility
Flagellar hook protein FlgE	TAN_78_316	9126	7936	-	Motility
Chemotaxis regulator - transmits chemoreceptor signals to flagelllar motor components CheY	TAN_78_137	18452	19366	+	Motility
Chemotaxis regulator - transmits chemoreceptor signals to flagelllar motor components CheY	TAN_78_287	9644	11557	+	Motility
Flagellar motor switch protein FliN	TAN_78_201	1157	675	-	Motility
Flagellum-specific ATP synthase FliI	TAN_78_201	8803	7445	-	Motility
Flagellar synthesis regulator FleN	TAN_78_30	19982	19155	-	Motility
Flagellar biosynthesis protein FliR	TAN_78_30	26228	25452	-	Motility
Flagellar assembly protein FliH	TAN_78_201	9569	8793	-	Motility
Sodium-type flagellar protein motY precursor	TAN_78_96	7236	6343	-	Motility
Flagellar hook-basal body complex protein FliE	TAN_78_201	12845	12396	-	Motility
Nitrate/nitrite transporter	TAN_78_105	1	684	+	Nitrate reduction
Nitrate reductase cytochrome c550-type subunit	TAN_78_125	20813	21301	+	Nitrate reduction
Nitrate/nitrite response regulator protein	TAN_78_136	455	1063	+	Nitrate reduction
Response regulator NasT	TAN_78_156	11318	12619	+	Nitrate reduction
Nitrite reductase [NAD(P)H] small subunit (EC 1.7.1.4)	TAN_78_204	4671	5000	+	Nitrate reduction
Response regulator NasT	TAN_78_58	26797	27375	+	Nitrate reduction
Respiratory nitrate reductase gamma chain (EC 1.7.99.4)	TAN_78_105	6850	7641	+	Nitrate reduction
Anaerobic nitric oxide reductase transcription regulator NorR	TAN_78_50	8114	9640	+	Nitrate reduction
Periplasmic nitrate reductase precursor (EC 1.7.99.4)	TAN_78_125	18297	20801	+	Nitrate reduction
Nitrate ABC transporter, ATP-binding protein	TAN_78_156	15217	15999	+	Nitrate reduction
Nitrite reductase [NAD(P)H] large subunit (EC 1.7.1.4)	TAN_78_204	2182	4647	+	Nitrate reduction
Nitrate/nitrite transporter	TAN_78_58	30286	31497	+	Nitrate reduction
periplasmic diheme c-type cytochrome, NapB	TAN_78_125	18043	18297	+	Nitrate reduction
Respiratory nitrate reductase beta chain (EC 1.7.99.4)	TAN_78_105	4560	6098	+	Nitrate reduction
Respiratory nitrate reductase delta chain (EC 1.7.99.4)	TAN_78_105	6102	6857	+	Nitrate reduction
Nitrate/nitrite sensor protein (EC 2.7.3.-)	TAN_78_136	97	477	+	Nitrate reduction
Nitrate ABC transporter, nitrate-binding protein	TAN_78_156	12978	14309	+	Nitrate reduction
Nitrate/nitrite sensor protein (EC 2.7.3.-)	TAN_78_193	5901	4639	-	Nitrate reduction
Nitrate ABC transporter, nitrate-binding protein	TAN_78_58	25566	26783	+	Nitrate reduction
Nitrite transporter from formate/nitrite family	TAN_78_173	5152	4490	-	Nitrate reduction
Assimilatory nitrate reductase large subunit (EC:1.7.99.4)	TAN_78_204	5043	7748	+	Nitrate reduction

Respiratory nitrate reductase alpha chain (EC 1.7.99.4)	TAN_78_105	790	4548	+	Nitrate reduction
Nitric oxide-dependent regulator DnrN or NorA	TAN_78_50	7968	7252	-	Nitrate reduction
Nitric oxide -responding transcriptional regulator Dnr (Crp/Fnr family)	TAN_78_50	6602	5919	-	Nitrate reduction
Nitric oxide reductase activation protein NorD	TAN_78_318	5040	7046	+	Nitrate reduction
Nitrous oxide reductase maturation protein NosD	TAN_78_111	19187	17880	-	Nitrate reduction
Nitrous oxide reductase maturation protein NosR	TAN_78_111	23033	21282	-	Nitrate reduction
Nitric-oxide reductase subunit B (EC 1.7.99.7)	TAN_78_50	2055	3479	+	Nitrate reduction
Nitrous oxide reductase maturation protein NosF (ATPase)	TAN_78_111	17883	16957	-	Nitrate reduction
Copper-containing nitrite reductase (EC 1.7.2.1)	TAN_78_318	413	1792	+	Nitrate reduction
Nitric-oxide reductase subunit B (EC 1.7.99.7)	TAN_78_318	2867	4228	+	Nitrate reduction
Nitrous oxide reductase maturation transmembrane protein NosY	TAN_78_111	16960	16130	-	Nitrate reduction
Nitric-oxide reductase subunit C (EC 1.7.99.7)	TAN_78_318	2016	2810	+	Nitrate reduction
Nitric-oxide reductase subunit C (EC 1.7.99.7)	TAN_78_50	1571	2011	+	Nitrate reduction
Nitric oxide reductase activation protein NorE	TAN_78_111	4333	4920	+	Nitrate reduction
Nitrate/nitrite transporter NarK	TAN_78_89	2359	863	-	Nitrate reduction
Nitrous-oxide reductase (EC 1.7.99.6)	TAN_78_111	21214	19301	-	Nitrate reduction
ubiquinol cytochrome C oxidoreductase, cytochrome C1 subunit	TAN_78_252	24766	24266	-	Oxygen respiration
ABC transporter involved in cytochrome c biogenesis, CcmB subunit	TAN_78_30	6214	5543	-	Oxygen respiration
Type cbb3 cytochrome oxidase biogenesis protein CcoS, involved in heme b insertion	TAN_78_166	40755	40549	-	Oxygen respiration
Cytochrome oxidase biogenesis protein Cox11-CtaG, copper delivery to Cox1	TAN_78_18	20694	20119	-	Oxygen respiration
Cytochrome c oxidase subunit CcoO (EC 1.9.3.1)	TAN_78_166	46651	46448	-	Oxygen respiration
Cytochrome c oxidase subunit CcoP (EC 1.9.3.1)	TAN_78_78	2722	1964	-	Oxygen respiration
Cytochrome c-type biogenesis protein CcmE, heme chaperone	TAN_78_30	4530	4063	-	Oxygen respiration
Putative analog of CcoH, COG3198	TAN_78_166	43776	43276	-	Oxygen respiration
Cytochrome oxidase biogenesis protein Sco1/SenC/PrrC, putative copper metallochaperone	TAN_78_18	15453	14818	-	Oxygen respiration
Cytochrome oxidase biogenesis protein Sco1/SenC/PrrC, putative copper metallochaperone	TAN_78_18	16530	15631	-	Oxygen respiration
Cytochrome c oxidase polypeptide II (EC 1.9.3.1)	TAN_78_18	23518	22409	-	Oxygen respiration
Cytochrome c oxidase subunit CcoP (EC 1.9.3.1)	TAN_78_166	46255	45338	-	Oxygen respiration
Type cbb3 cytochrome oxidase biogenesis protein CcoI; Copper-translocating P-type ATPase (EC 3.6.3.4)	TAN_78_166	43178	40848	-	Oxygen respiration

Supplementary Table 7. Methodological parameters for determining N_2 fixation rates during the Sep/Oct 2017 campaign. SD standards: standard deviation of all internal standards during the respective mass spectrometer run. The calculated limit of detection (LOD) was defined as $\delta^{15}N = \delta^{15}N_{control}$ + 3*standard deviation($\delta^{15}N_{standards}$). The applied LOD was conservatively defined as $\delta^{15}N_{control}$ + 4 ‰ (minimum change; Montoya et al.[32]). Treatment samples >LOD are indicated in dark green and treatment samples <LOD in light green.

station	sample	depth	$\delta^{15}N$	atom % sample	mass N	atom % $^{15-15}N_{2\ (aq.)}$	LOD (calculated)	$\Delta\delta^{15}N$ (sample-LOD)	$\Delta\delta^{15}N$ (sample-control)	volume filtered	incubation time	N fixation rate
			‰	%	µg	%	‰	‰	‰	L	d	nM N d^{-1}
2	1	1	1.4	0.3670	16.9	5.65	1.4	0.0	0.3			
	2		1.1	0.3669	15.0	6.11	1.4	-0.3	0.0			
	1	40	-0.6	0.3663	33.3	2.60	-0.7	0.1	0.4			
	2		0.6	0.3667	35.6	6.45	-0.7	1.3	1.6			
	control	1	1.1	0.3669	21.2							
		40	-1.0	0.3661	28.0							
	SD standards		0.1	0.00004								
	t0	1	0.5									
		40	0.4									
9	1	1	72.6	0.3930	22.4	6.67	2.6	70.0	70.3	3.7	1.05	**2.3**
	2		13.8	0.3715	19.5	3.65	2.6	11.2	11.5	3.7	1.03	**1.2**
	1	40	2.1	0.3672	22.3	6.09	0.8	1.4	1.7			
	2		0.4	0.3666	25.6	6.63	0.8	-0.3	0.0			
	control	1	2.3	0.3673	20.5							
		40	0.5	0.3666	21.7							
	SD standards		0.1	0.00004								
	t0	1	0.6									
		40	0.9									

Supplementary Table S8. Methodological parameters for determining N_2 fixation rates during the Apr/May 2018 campaign. SD standards: standard deviation of all internal standards during the respective mass spectrometer run. The calculated limit of detection (LOD) was defined as $\delta^{15}N = \delta^{15}N_{control}$ + 3*standard deviation($\delta^{15}N_{standards}$). The applied LOD was conservatively defined as $\delta^{15}N_{control}$ + 4 ‰ (minimum change; Montoya et al.[32]). Treatment samples >LOD are indicated in dark green and treatment samples <LOD in light green.

station	sample	depth	batch	$\delta^{15}N$	atom % sample	mass N	atom % $^{15\text{-}15}N_2$ (aq.)	LOD (calculated)	$\Delta\delta^{15}N$ (sample-LOD)	$\Delta\delta^{15}N$ (sample-control)	volume filtered	incubation time	N fixation rate
				‰	%	µg	%	‰	‰	‰	L	d	nM N d^{-1}
	1	1	190111	16.2	0.3779	57.2	3.57	-0.4	16.6	17.7	4.57	1.00	**1.5**
	2		190321	23.5	0.3800	46.1	4.37	-0.7	24.3	25.0	4.57	1.00	**1.4**
	1	20	190111	77.1	0.4007	51.9	4.95	-2.4	79.5	80.5	4.57	1.00	**5.5**
	2		190321	168.1	0.4359	54.3	5.16	-2.7	170.8	171.5	3.83	1.02	**13.7**
	1	50	190111	11.3	0.3749	34.6	4.71	1.9	9.4	10.5	3.74	1.03	**0.6**
	2		190321	8.5	0.3738	50.8	6.15	1.6	7.0	7.7	4.57	1.04	**0.4**
	1	144	190111	24.5	0.3799	26.7	3.94	8.4	16.1	17.1	4.57	1.03	**0.8**
	2		190321	24.5	0.3792	28.5	4.23	8.1	16.4	17.1	4.57	1.05	**0.7**
2	1	156	190111	-6.8	0.3666	30.5	3.62	-8.7	1.9	2.9			
	2		190321	-1.1	0.3699	42.6	4.33	0.7	-1.8	8.6			
	control	156	190321	-9.8	0.3669	37.1							
		144	190321	7.4	0.3728	27.5							
		20	190321	-3.4	0.3696	49.9							
		1	190321	-1.4	0.3725	56.4							
		50	190321	0.9	0.3708	56.4							
	SD standards		190111	0.3	0.0001								
			190321	0.2	0.0005								
	1	1	190124	220.3	0.4507	28.1	1.77	1.8	218.5	219.3	4.57	1.02	**24.4**
	2		190228	266.8	0.4683	26.1	2.19	1.9	264.9	265.8	4.57	1.02	**21.3**
	1	43	190124	-3.4	0.3706	40.5	0.60	0.5	-3.9	-3.2			
	2		190228	-1.0	0.3702	52.4	1.58	0.7	-1.7	-0.8			
	1	60	190124	-0.2	0.3710	42.7	3.21	2.0	-2.1	-1.4			
	2		190228	-2.0	0.3714	45.0	0.61	2.1	-4.2	-3.2			
	1	100	190124	5.3	0.3707	17.6	2.03	4.0	1.3	2.0			
	2		190228	1.5	0.3679	14.8	0.03	4.1	-2.7	-1.7			

	1	140	190124	-0.3	0.3706	40.3	0.02	0.6	-0.9	-0.1			
	2		190228	4.0	0.3723	48.5	0.16	0.8	3.2	4.2	4.25	1.05	-6.8
4	control	140	190124	-0.2	0.3705	31.0							
		100	190124	3.2	0.3710	23.7							
		60	190124	1.2	0.3713	47.4							
		43	190124	-0.2	0.3709	55.8							
		1	190124	1.0	0.3713	33.1							
	SD standards		190124	0.3	0.0002								
			190228	0.3	0.0006								
	t0	1		0.3									
		43		-1.7									
		60		7.2									
		100		4.1									
		140		10.8									
	1	1	190111	28.2	0.3818	35.6	5.02	0.3	27.9	29.0	4.57	1.01	**1.4**
	2		190321	30.5	0.3822	44.6	4.54	0.0	30.6	31.3	4.57	1.01	**2.0**
	1	43	190111	16.1	0.3717	18.6	10.43	-0.8	16.9	17.9	4.57	1.01	**0.02**
	2		190321	136.0	0.4214	78.5	6.22	-1.1	137.1	137.8	4.57	1.04	**10.1**
	1	63	190111	2.1	0.3689	21.3	6.76	-4.4	6.5	7.5	4.57	1.04	-0.4
	2		190321	1.4	0.3704	26.7	7.56	-4.7	6.2	6.9	4.57	1.03	-0.3
	1	85	190111	0.2	0.3690	17.3	7.28	-0.8	1.0	2.0			
	2		190321	3.2	0.3705	19.7	6.59	-1.2	4.3	5.0	4.57	1.07	0.1
	1	135	181206	-1.9	0.3708	15.9	6.40	0.9	-2.7	-2.6			
	2		181206	0.7	0.3722	17.5	6.69	0.9	-0.1	0.1			
6	control	135	181206	0.7	0.3721	11.9							
		85	190111	-1.9	0.3673	21.2							
		63	190111	-5.5	0.3760	11.4							
		43	190111	-1.8	0.3711	37.9							
		1	190111	-0.8	0.3703	44.7							
	SD standards		190111	0.3	0.0001								
			190321	0.2	0.0005								
			181206	0.1	0.0001								
	t0	1		1.2									

		43		-1.3									
		63		1.2									
		85		3.0									
		135		3.7									
	1	1	191004	6.6	0.3760	33.6	7.66	0.6	6.0	4.6	4.57	1.02	**0.2**
	2		191004	14.0	0.3767	35.3	7.40	2.9	11.1	12.0	4.57	1.02	**0.3**
	1	43	191004	45.9	0.3874	48.1	7.33	2.7	43.3	44.1	4.57	1.02	**1.6**
	2		191004	11.3	0.3785	44.4	7.47	2.7	8.7	9.5	4.55	1.04	**0.6**
	1	62	191004	8.1	0.3769	31.3	7.35	0.9	7.3	8.1	4.57	1.05	**0.2**
	1	85	191004	11.4	0.3762	25.6	11.28	0.4	11.0	11.8	4.57	1.06	**0.1**
	2		191004	9.3	0.3751	19.1	9.97	0.4	9.0	9.8	4.55	1.07	**0.1**
7	1	150	191004	10.3	0.3754	17.6	9.84	1.4	8.9	9.7	4.57	1.08	**0.1**
	2		191004	13.9	0.3768	16.1	8.34	1.4	12.5	13.3	4.57	1.08	**0.1**
		1	191004	2.0	0.3732	29.8							
		43	191004	1.8	0.3723	18.1							
	control	62	191004	0.0	0.3732	34.2							
		85	191004	-0.5	0.3723	17.7							
		150	191004	0.5	0.3719	18.0							
	SD standards		191004	0.3	0.0009								
	1	1	190124	29.1	0.3825	38.8	8.48	1.0	28.1	29.0	4.555	1.01	**0.8**
	2		190228	67.6	0.3957	43.0	7.53	0.9	66.7	67.5	4.555	1.01	**2.2**
	1	28	190124	26.9	0.3799	25.1	7.77	0.1	26.9	27.8	4.555	1.03	**0.5**
	2		190228	115.8	0.4141	57.1	7.45	-0.1	115.9	116.6	4.555	1.00	**5.4**
	1	60	190124	2.3	0.3710	27.7	6.89	-0.5	2.8	3.7			
	2		190228	2.4	0.3710	38.9	6.95	-0.7	3.1	3.8			
	1	113	190124	2.3	0.3711	20.2	6.96	1.2	1.1	2.0			
	2		190228	-0.4	0.3701	24.5	9.37	1.1	-1.5	-0.7			
	1	150	190124	1.3	0.3708	36.4	6.76	2.1	-0.8	0.1			
	2		190228	0.5	0.3708	26.6	6.57	1.9	-1.4	-0.6			
9		150	190228	1.2	0.3707	28.2							
		113	190228	0.3	0.3690	23.6							
	control	60	190228	-1.4	0.3697	26.7							
		28	190228	-0.9	0.3710	53.5							

		1	190228	0.1	0.3720	53.6
	SD standards		190124	0.3	0.0002	
			190228	0.3	0.0006	
	t0	1		2.4		
		28		1.5		
		60		3.9		
		113		4.7		
		150		2.4		

Supplementary References

1. Schubert, C. J. *et al.* Anaerobic ammonium oxidation in a tropical freshwater system (Lake Tanganyika). *Environ. Microbiol.* **8**, 1857–1863 (2006).
2. Callbeck, C. M., Ehrenfels, B., Baumann, K. B. L., Wehrli, B. & Schubert, C. J. Anoxic chlorophyll maximum enhances local organic matter remineralization and nitrogen loss in Lake Tanganyika. *Nat. Commun.* **12**, (2021).
3. Dore, J. E., Letelier, R. M., Church, M. J., Lukas, R. & Karl, D. M. Summer phytoplankton blooms in the oligotrophic North Pacific Subtropical Gyre: Historical perspective and recent observations. *Prog. Oceanogr.* **76**, 2–38 (2008).
4. Follett, C. L., Dutkiewicz, S., Karl, D. M., Inomura, K. & Follows, M. J. Seasonal resource conditions favor a summertime increase in North Pacific diatom-diazotroph associations. *ISME J.* **12**, 1543–1557 (2018).
5. McGillicuddy, D. J. Do Trichodesmium spp. populations in the North Atlantic export most of the nitrogen they fix? *Global Biogeochem. Cycles* **28**, 103–114 (2014).
6. Kalvelage, T. *et al.* Nitrogen cycling driven by organic matter export in the South Pacific oxygen minimum zone. *Nat. Geosci.* **6**, 228–234 (2013).
7. Fuchsman, C. A., Paul, B., Staley, J. T., Yakushev, E. V. & Murray, J. W. Detection of Transient Denitrification During a High Organic Matter Event in the Black Sea. *Global Biogeochem. Cycles* **33**, 143–162 (2019).
8. Dalsgaard, T., Thamdrup, B., Farías, L. & Revsbech, N. P. Anammox and denitrification in the oxygen minimum zone of the eastern South Pacific. *Limnol. Oceanogr.* **57**, 1331–1346 (2012).
9. Erb, T. J. Carboxylases in Natural and Synthetic Microbial Pathways. *Appl Env. Microbiol* **77**, 8466–8477 (2011).
10. Pontiller, B., Martínez-García, S., Lundin, D. & Pinhassi, J. Labile Dissolved Organic Matter Compound Characteristics Select for Divergence in Marine Bacterial Activity and Transcription. *Front. Microbiol.* **11**, 588778 (2020).
11. Tang, K., Jiao, N., Liu, K., Zhang, Y. & Li, S. Distribution and functions of TonB-dependent transporters in marine bacteria and environments: implications for dissolved organic matter utilization. *PLoS One* **7**, e41204–e41204 (2012).
12. Ehrenfels, B. *et al.* Diazotrophic cyanobacteria are associated with a low nitrate resupply to surface waters in Lake Tanganyika. *Front. Environ. Sci.* **9**, 277 (2021).
13. Schmieder, R. & Edwards, R. Quality control and preprocessing of metagenomic datasets. *Bioinformatics* **27**, 863–864 (2011).
14. Menzel, P., Ng, K. L. & Krogh, A. Fast and sensitive taxonomic classification for metagenomics with Kaiju. *Nat. Commun.* **7**, 1–9 (2016).
15. Li, D., Liu, C. M., Luo, R., Sadakane, K. & Lam, T. W. MEGAHIT: An Ultra-Fast Single-Node Solution for Large and Complex Metagenomics Assembly via Succinct de Bruijn Graph. *Bioinformatics* **31**, 1674–1676 (2015).
16. Bushnell, B. *BBMap: a fast, accurate, splice-aware aligner (No. LBNL-7065E)*. (Lawrence Berkeley National Lab, Berkeley, USA. https://sourceforge.net/projects/bbmap/, 2014).
17. Tarasov, A., Vilella, A. J., Cuppen, E., Nijman, I. J. & Prins, P. Sambamba: fast processing of NGS alignment formats. *Bioinformatics* **31**, 2032–2034 (2015).
18. Li, H. *et al.* The Sequence Alignment/Map format and SAMtools. *Bioinformatics* **25**, 2078–2079 (2009).
19. Liao, Y., Smyth, G. K. & Shi, W. featureCounts: an efficient general purpose program for assigning sequence reads to genomic features. *Bioinformatics* **30**, 923–930 (2014).
20. Seemann, T. Prokka: rapid prokaryotic genome annotation. *Bioinformatics* **30**, 2068–2069 (2014).
21. Uritskiy, G. V., Diruggiero, J. & Taylor, J. MetaWRAP - A Flexible pipeline for genome-resolved

metagenomic data analysis. *Microbiome* **6**, 1–13 (2018).
22. Parks, D. H., Imelfort, M., Skennerton, C. T., Hugenholtz, P. & Tyson, G. W. CheckM: Assessing the Quality of Microbial Genomes recovered from isolates, single cells, and metagenomes. *Genome Res.* **25**, 1043–1055 (2015).
23. Bowers, R. M. *et al.* Minimum Information about a Single Amplified Genome (MISAG) and a Metagenome-Assembled Genome (MIMAG) of Bacteria and Archaea. *Nat. Biotechnol.* **35**, 725–731 (2017).
24. Chaumeil, P. A., Mussig, A. J., Hugenholtz, P. & Parks, D. H. GTDB-Tk: A Toolkit to classify genomes with the genome taxonomy database. *Bioinformatics* **36**, 1925–1927 (2020).
25. Kumar, S., Stecher, G., Li, M., Knyaz, C. & Tamura, K. MEGA X: Molecular evolutionary genetics analysis across computing platforms. *Mol. Biol. Evol.* **35**, 1547–1549 (2018).
26. Granger, J. & Sigman, D. M. Removal of nitrite with sulfamic acid for nitrate N and O isotope analysis with the denitrifier method. *Rapid Commun. Mass Spectrom.* **23**, 3753–3762 (2009).
27. Sigman, D. M. *et al.* A bacterial method for the nitrogen isotopic analysis of nitrate in seawater and freshwater. *Anal. Chem.* **73**, 4145–4153 (2001).
28. Casciotti, K. L., Sigman, D. M., Hastings, M. G., Böhlke, J. K. & Hilkert, A. Measurement of the oxygen isotopic composition of nitrate in seawater and freshwater using the denitrifier method. *Anal. Chem.* **74**, 4905–4912 (2002).
29. McIlvin, M. R. & Casciotti, K. L. Technical updates to the bacterial method for nitrate isotopic analyses. *Anal. Chem.* **83**, 1850–1856 (2011).
30. Houlton, B. Z., Sigman, D. M., Schuur, E. A. G. & Hedin, L. O. A climate-driven switch in plant nitrogen acquisition within tropical forest communities. *Proc. Natl. Acad. Sci.* **104**, 8902–8906 (2007).
31. Bourbonnais, A., Lehmann, M. F., Butterfield, D. A. & Juniper, S. K. Subseafloor nitrogen transformations in diffuse hydrothermal vent fluids of the Juan de Fuca Ridge evidenced by the isotopic composition of nitrate and ammonium. *Geochemistry, Geophys. geosystems* **13**, (2012).
32. Montoya, J. P., Voss, M., Kahler, P. & Capone, D. G. A simple high-precision, high-sensitivity tracer assay for N_2 fixation. *Appl. Environ. Microbiol.* **62**, 986–993 (1996).
