# Supplementary material for: Hydrodynamic regimes modulate nitrogen fixation and the mode of diazotrophy in Lake Tanganyika
Source: Nat Commun. 2023 Oct 18;14:6591. doi: 10.1038/s41467-023-42391-3 (PMC10584864; doi:10.1038/s41467-023-42391-3)
Supplement: Supplementary file 3 — Reporting Summary [file 41467_2023_42391_MOESM3_ESM.pdf]

nature portfolio

Corresponding author(s): Cameron Callbeck

Last updated by author(s): Sep 20, 2023

Reporting Summary

Nature Portfolio wishes to improve the reproducibility of the work that we publish. This form provides structure for consistency and transparency in reporting. For further information on Nature Portfolio policies, see our Editorial Policies and the Editorial Policy Checklist.

Statistics

For all statistical analyses, confirm that the following items are present in the figure legend, table legend, main text, or Methods section.

n/a	Confirmed	
[ ]	[x]	The exact sample size (*n*) for each experimental group/condition, given as a discrete number and unit of measurement
[ ]	[x]	A statement on whether measurements were taken from distinct samples or whether the same sample was measured repeatedly
[ ]	[x]	The statistical test(s) used AND whether they are one- or two-sided<br>*Only common tests should be described solely by name; describe more complex techniques in the Methods section.*
[x]	[ ]	A description of all covariates tested
[x]	[ ]	A description of any assumptions or corrections, such as tests of normality and adjustment for multiple comparisons
[ ]	[x]	A full description of the statistical parameters including central tendency (e.g. means) or other basic estimates (e.g. regression coefficient) AND variation (e.g. standard deviation) or associated estimates of uncertainty (e.g. confidence intervals)
[ ]	[x]	For null hypothesis testing, the test statistic (e.g. *F*, *t*, *r*) with confidence intervals, effect sizes, degrees of freedom and *P* value noted<br>*Give P values as exact values whenever suitable.*
[x]	[ ]	For Bayesian analysis, information on the choice of priors and Markov chain Monte Carlo settings
[x]	[ ]	For hierarchical and complex designs, identification of the appropriate level for tests and full reporting of outcomes
[x]	[ ]	Estimates of effect sizes (e.g. Cohen's *d*, Pearson's *r*), indicating how they were calculated

Our web collection on statistics for biologists contains articles on many of the points above.

Software and code

Policy information about availability of computer code

Data collection	Sentinel-3 OLCI data (v.1.3 Copernicus Global Land Service) - public satellite data
Data analysis	Microsoft Excel 2019, GraphPad Prism 10 (v10.0.2), Ocean Data View (ODV 5.6.5); FastQC (v0.12.1); Megahit (v1.2.9); metaWRAP (v1.3); CheckM (v1.1.6); coverM (v0.2.0); Sentinel-3 OLCI data; Ion Vantage (Isoprime v1.7.3.0), prokka v1.13, MEGAX (version 11.0.13), SAMtools (version 1.18), sambamba (GPL v2+), Kaiju (version 1.9.2).

For manuscripts utilizing custom algorithms or software that are central to the research but not yet described in published literature, software must be made available to editors and reviewers. We strongly encourage code deposition in a community repository (e.g. GitHub). See the Nature Portfolio guidelines for submitting code & software for further information.

Data

Policy information about availability of data

All manuscripts must include a data availability statement. This statement should provide the following information, where applicable:

- Accession codes, unique identifiers, or web links for publicly available datasets
- A description of any restrictions on data availability
- For clinical datasets or third party data, please ensure that the statement adheres to our policy

The physicochemical data in this study have been deposited in the ETH Zurich Research Collection: https://www.research-collection.ethz.ch/handle/20.500.11850/418479. The Sentinel-3 OLCI satellite data used in this study can be obtained via the following public database: https://sentinels.copernicus.eu/web/sentinel/missions/sentinel-3. The metagenomic data in this study have been deposited in the NCBI database under the accession code

PRJNA675607 (https://www.ncbi.nlm.nih.gov/sra/?term=PRJNA675607). Data pertaining to N2 fixation measurements are available in the Supplementary Tables 7 and 8.

Research involving human participants, their data, or biological material

Policy information about studies with human participants or human data. See also policy information about sex, gender (identity/presentation), and sexual orientation and race, ethnicity and racism.

Reporting on sex and gender	not applicable
Reporting on race, ethnicity, or other socially relevant groupings	not applicable
Population characteristics	not applicable
Recruitment	not applicable
Ethics oversight	not applicable

Note that full information on the approval of the study protocol must also be provided in the manuscript.

Field-specific reporting

Please select the one below that is the best fit for your research. If you are not sure, read the appropriate sections before making your selection.

☐ Life sciences ☐ Behavioural & social sciences ☒ Ecological, evolutionary & environmental sciences

For a reference copy of the document with all sections, see nature.com/documents/nr-reporting-summary-flat.pdf

Ecological, evolutionary & environmental sciences study design

All studies must disclose on these points even when the disclosure is negative.

Study description	Here, we examined the distribution of nitrogen fixation in Lake Tanganyika (Tanzania) – a model system with well-defined hydrodynamic regimes. Specifically, we explored the distribution of N2 fixation and associated diazotrophs under contrasting hydrodynamic regimes across a north-south transect in Lake Tanganyika. We obtained water column samples at various depths and performed stable isotope applications (in duplicates) and extracted DNA for metagenomic analyses on collected water.
Research sample	Water was collected from Lake Tanganyika to determine both the rates of N2 fixation and the key microbial N2 fixing diazotrophs involved. Depths were selected according to previous studies in this Lake, with sample collection occurring in both the near-surface waters and in the anoxic zone (where it is suspected to harbour active N2 fixing microbial communities). The investigated diazotrophs included Aquabacterium, Pseudomonas, Dolichospermum, and Chlorobium, which were identified (based on DNA sequencing) in our study to contain a key gene involved in N2 fixation (nifH). This study did not rely on existing dataset, data collected here is original.
Sampling strategy	No sample-size calculation was done. Samples size was determined by how many samples were need to resolve biogeochemical gradients in the water column. The number of samples collected is also the maximum that can be carried out within a defined sampling period (on-station time constraints). For example, N2 fixation rates were measured at five depths, which is the maximum number of samples that could be taken at a particular station (one station per day over a 1-1.5 week period). Moreover, 15N incubations to determine N2 fixation rates were performed in two independent biological replicates per depth, which is standard procedure for environmental biogeochemical rate measurements (e.g. GroBkopf et al. 2012, https://doi.org/10.1038/nature11338; Bonnet et al. 2013, https:// doi.org/10.1371/journal.pone.0081265; Martinez-Perez et al. 2016, https://doi.org/10.1038/nmicrobiol.2016.163).
Data collection	A number of co-authors were involved in data collection: Benedikt Ehrenfels, Kathrin Baumann, Athanasio S. Mbonde, Daniel Odermatt, Cameron M. Callbeck. In situ water profiles were measured using a CTD system and recorded with an attached laptop (B.E.). Water sampling, incubation set up, water treatment and filtration details were recorded in a labbook (B.E., K.B., A.S.M., C.M.C.). Cyanobacterial cell counts were counted under a microscope and recorded in a labbook (A.S.M.). 15N isotope analyses was measured with a mass spectrometer and the data stored on a computer (B.E., C.M.C). Satellite remote sensing data of chlorophyll was obtained via public databases and stored on a computer (D.O.).
Timing and spatial scale	Sampling campaigns took place in September/October-2017 (28 September - 8 October), April/May-2018 (27 April - 7 May) and April/May-2019 (26 April - 1 May). A North-South lake transect was performed (spanning from 4.5 down to 9 degrees South latitude, with stations sampled at 0.5 degree resolution in-between). CTD profiles, and nutrients were taken at each station from 0 to 200 m depth (15 depths in-between), while samples for DNA extraction (metagenomics analysis, 5-6 depths between 0-200 m) and N2 fixation experiments were carried out at every second station (five incubation depths from 0-200 m, with the main focus on resolving the upper oxic and anoxic zones). N2 incubation experiments were terminated on-site after a 24 hour period.
Data exclusions	No data was excluded from the 2018 and 2017 campaigns (main campaigns in the study). However, N2 fixation rates from the 2019 campaign were not included in the study due to methodological issues.
Reproducibility	All isotope measurements were reproducible from duplicate measurements. In some cases, where a limited sample volume is

	available for an analysis and/or the cost to reproduce the measurement is not feasible (i.e., metagenomic DNA sequencing) then reproducibility was not assessed.
Randomization	Not performed on site during the sample collection. However, mass spectrometry samples (to determine rates of N2 fixation) were measured in a randomized sequence and identified through a code.
Blinding	Not performed during the sample collection. However, the person performing the mass spectrometry measurements (to determine rates of N2 fixation) had no prior knowledge of the sample identity, which were later identified via a unique code.

Did the study involve field work? ☒ Yes ☐ No

Field work, collection and transport

Field conditions	Samples were taken in a tropical lake, hence, temperatures averaged 30 degrees with periodic thunderstorms.
Location	Samples were taken along the length of the lake from 4 to 9 degrees south (latitude)
Access & import/export	We worked with our host institute to export and import samples responsibly. All permits were obtained through the host institute.<br>Director of host institute: Ismael A. Kimirei<br>TAFIRI, Tanzania Fisheries Research Institute, Kigoma, Tanzania.<br>TAFIRI, Tanzania Fisheries Research Institute, Dar es Salaam, Tanzania.
Disturbance	No disturbances to report.

Reporting for specific materials, systems and methods

We require information from authors about some types of materials, experimental systems and methods used in many studies. Here, indicate whether each material, system or method listed is relevant to your study. If you are not sure if a list item applies to your research, read the appropriate section before selecting a response.

Materials & experimental systems

n/a	Involved in the study
☒	☐ Antibodies
☒	☐ Eukaryotic cell lines
☒	☐ Palaeontology and archaeology
☒	☐ Animals and other organisms
☒	☐ Clinical data
☒	☐ Dual use research of concern
☒	☐ Plants

Methods

n/a	Involved in the study
☒	☐ ChIP-seq
☒	☐ Flow cytometry
☒	☐ MRI-based neuroimaging
